# Tuning charge transport by manipulating concentration dependent single-molecule absorption configurations

**DOI:** 10.1016/j.isci.2024.109292

**Published:** 2024-02-20

**Authors:** Xia Long, Wangping Xu, Tingting Duan, Liyan Lin, Yandong Guo, Xiaohong Yan, Juexian Cao, Yong Hu

**Affiliations:** 1 Hunan Institute of Advanced Sensing and Information Technology, Xiangtan University, Xiangtan, China; 2 College of Electronic and Optical Engineering, Nanjing University of Posts and Telecommunications, Nanjing, China

**Keywords:** transport phenomena, molecular electrochemistry

## Abstract

Understanding and tuning charge transport in molecular junctions is pivotal for crafting molecular devices with tailored functionalities. Here, we report a novel approach to manipulate the absorption configuration within a 4,4′-bipyridine (4,4′-BPY) molecular junction, utilizing the scanning tunneling microscope break junction technique in a concentration-dependent manner. Single-molecule conductance measurements demonstrate that the molecular junctions exhibit a significant concentration dependence, with a transition from high conductance (HC) to low conductance (LC) states as the concentration decreases. Moreover, we identified an additional conductance state in the molecular junctions besides already known HC and LC states. Flicker noise analysis and theoretical calculations provided valuable insights into the underlying charge transport mechanisms and single-molecule absorption configurations concerning varying concentrations. These findings contribute to a fundamental comprehension of charge transport in concentration-dependent molecular junctions. Furthermore, they offer promising prospects for controlling single-molecule adsorption configurations, thereby paving the way for future molecular devices.

## Highlights

• Manipulation of single-molecule absorption configurations by concentration
• Tunability of charge transport within molecular junction at the single-molecule scale
• An additional conductance state is observed besides already known high and low states

## Graphical abstract

**Figure:**
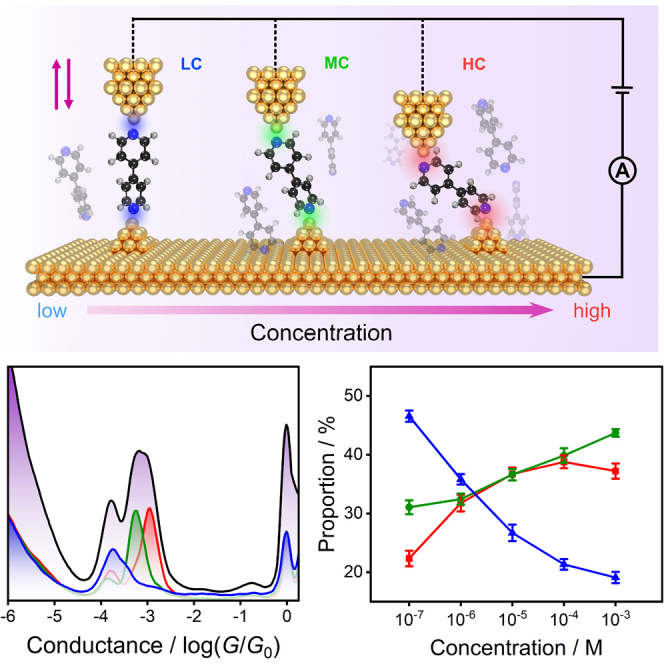

## Introduction

Single-molecule devices have attracted significant interest in the field of molecular electronics due to their novel quantum phenomena and the extreme miniaturization of electronic devices.^1^^,^^2^^,^^3^ Molecular junctions offer a compelling avenue for constructing molecular devices.^4^^,^^5^^,^^6^^,^^7^^,^^8^^,^^9^ It is crucial to comprehend and manipulate charge transport in molecular junctions to design molecular devices with desired functionalities.^10^^,^^11^^,^^12^^,^^13^^,^^14^ Recently, external conditions such as electric field,^15^ pH,^16^ and electrochemical gating^17^ have been used to control the charge transport properties of molecular junctions, which affects the charge transport at the molecular junctions by altering the electronic structure of molecules. To fully understand the charge transport properties of molecular devices, not only the relationship between the intrinsic structure of the molecule and the charge transport properties but also the influence of intermolecular interactions should be considered. At present, the charge transport properties of intermolecular interactions have been widely studied, such as hydrogen bonding^18^^,^^19^^,^^20^ and π-π stacking interactions.^21^^,^^22^ However, the potential implications of concentration, a pivotal factor governing intermolecular interaction with the interface, are frequently underestimated. Hence, it is essential to study the influence of single-molecule absorption configurations caused by the concentration gradients on charge transport within molecular junctions.

Dipyridyl molecular junctions have double-peak conductance signatures, which makes them promising for mechanically activated switches.^23^^,^^24^ The 4,4′-bipyridine (4,4′-BPY) molecular junction is a typical example, it has high and low conductance (LC) states caused by the varying coupling strengths between the molecule and the gold substrate. The high conductance (HC) state is a result of the π-bond of the pyridine ring binding to the gold substrate (π-binding), while the LC state is caused by the N atom binding to the gold substrate (δ-binding).^8^ The current single-molecule measurement techniques^25^^,^^26^ such as mechanically controlled break junction (MCBJ), and scanning tunneling microscope break junction (STM-BJ), combined with theoretical calculation can be used to measure and explain the charge transport properties in molecular junctions.^27^^,^^28^ For instance, the electrochemical gating approach is used to control the charge transport by affecting the relative position between the frontier orbit of the molecule and the Fermi level of the electrode.^17^^,^^29^ Meanwhile, modification of the solvent can change the electronic state of the interface, thereby modulating the charge transport properties of the molecular junction.^30^ However, the effect of concentration on the charge transport of dipyridyl molecules remains challenging, due to the difficulties in characterizing single-molecule absorption configurations and probing charge transport at extremely low concentrations.

In this work, we apply the STM-BJ technique to experimentally probe the charge transport of dipyridyl junctions at different concentrations. Conductance measurements reveal that the concentration has an impact on the conductance of the molecular junction. With the aid of the clustering analysis technique, three distinct conductance states of high, medium, and LC were observed in the 4,4′-BPY molecular junction. In addition, it was also found that with the concentration decreased, the proportion of HC and medium conductance (MC) decreased, while the proportion of LC increased. This is because at high concentrations, the distances between molecules are small and the interactions are stronger, which has a great influence on the charge transport properties of the molecular junction. It was further confirmed by flicker noise spectral to probe the coupling at the metal-molecule interface. Finally, density functional theory (DFT) calculation and *ab initio* molecular dynamics (AIMD) simulation were employed to understand the origin of these three conductance states and to provide an interpretation of the influence of concentration on single-molecule absorption configurations, leading to the changes in charge transport.

## Results and discussion

### Single-molecule conductance measurements

Conductance measurements were performed using a home-built STM-BJ setup (Figure S1). For the two gold electrodes, one is the gold tip briefly in a flame to remove any possible contamination, while the other is the substrates coated with 200 nm gold by sputtering. A constant bias potential (V bias) of 100 mV was applied between the gold substrate and gold tip. The solution was then placed on the gold substrate and the distance between the gold tip and the substrate was controlled by piezo. When the tip contacts the gold substrate, the current (or conductance) of the system will change significantly, and then pull the tip upward too far away from the substrate. During the pulling process, the gold tip and substrate are connected with the amino groups in solution to form a molecular junction, and then the tip continues to move upward until the molecular junctions break. As the tip moves upward, the molecular junction breaks and then continues to move upward until the conductance is 10^−7^ *G*_0_, Where *G*_0_ = 2*e*^2^/*h* (*G*_0_ = 77.5 μS) is the quantum of conductance. The tip stops moving upward and starts moving down again to approach the substrate. In this way, more conductance and distance curves can be measured to determine the conductance of the molecular junction. Before each broken connection measurement, the substrate was immersed in ethanol for at least 2 h, followed by transfer to ultra-pure water for boiling. Then rinse with ethanol and ultra-pure water three times, and immerse the substrate in a piranha solution [V (H_2_O_2_): V (H_2_SO_4_) = 1:3] for about 4 h. It was then washed with new ultra-pure water for 5 min and placed in the drying box to dry at 120°C.

We measure the one-dimensional (1D) and two-dimensional (2D) conductance histograms of molecular junctions in solution (1,2,4-trichlorobenzene, TCB) without/with 4,4′-BPY (10^−3^ M), as illustrated in Figures S2 and S3. In contrast to the pure solvent, which does not exhibit a conductance peak (Figure S2), two conductance peaks of 4,4′-BPY can be seen clearly, and their positions in the 1D conductance histogram are close to 10^−3.0^*G*_0_ (77.5 nS, HC) and 10^−3.8^*G*_0_ (12.3 nS, LC), respectively, which is consistent with the previous report.^8^ The acknowledged geometric configurations of the two conductance states are shown in Figure S4, where the dihedral angle between the adsorbed pyridine ring and the surface is acute at HC. In this case, the aromatic ring in the molecule forms an π-bond with the gold substrate. Moreover, the LC is caused by the N atom of the molecule forming a δ-bond with the gold, and the molecule positioned upright between the gold electrodes. This fundamental reason underlies the observation of HC and LC in the measurement of the 4,4′-BPY molecular junctions.^8^^,^^31^ As a result, two different cloud atlas in the 2D conductance distance histogram (Figure S3B), which is caused by the different stretching states of the molecular junction.

**Figure 1:**
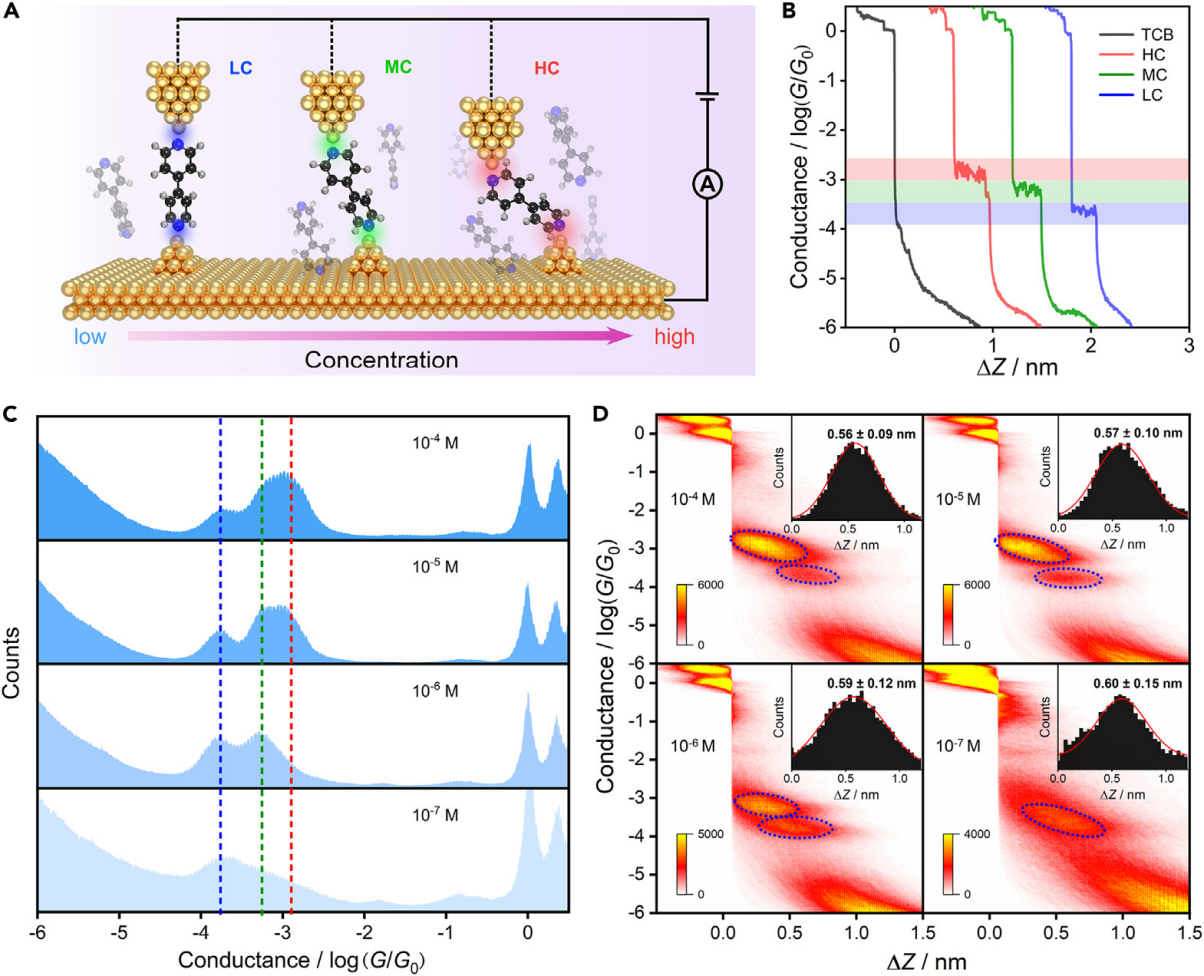
Conductance measurement results (A) STM-BJ schematic diagram of 4,4′-BPY molecular junctions. (B) Single conductance-distance traces of pure TCB (black) and the three conductance states of 4,4′-BPY molecular junctions, red, green, and blue represent HC, MC, and LC, respectively. (C) 1D and (D) 2D conductance histograms of 4,4′-BPY molecular junctions at 10^−4^ M (8,493 traces), 10^−5^ M (8,041 traces), 10^−6^ M (6,261 traces) and 10^−7^ M (7,929 traces) concentrations, the red, green, and blue dashed lines correspond to HC, MC, and LC, respectively. The insets are stretching distance distributions of molecular junctions ranging from 10^−0.3^*G*_0_ to 10^−4.5^*G*_0_, the lengths are determined by Gaussian fitting.

To investigate the effect of intermolecular interaction on the charge transport properties of the 4,4′-BPY molecular junction, we measured the conductance of the 4,4′-BPY molecular junction at concentrations ranging from 10^−4^ M to 10^−7^ M, which was obtained by successively diluting 10 times. Figure 1B shows some representative conductance-distance traces collected from over 8000 traces. The 1D conductance histograms at different concentrations (Figure 1C) indicate that when the concentration decreases, the position of the HC peak shifts to the left, and a shoulder peak appears at a concentration of 10^−5^ M, while the position of the LC peak remains constant. Based on this phenomenon, we postulate that the 4,4′-BPY molecular junction exhibits three different conductance states: HC, MC, and LC. The 2D conductance histograms at different concentrations are shown in Figure 1D. It is evident that as the concentration decreases, the intensity clouds corresponding to the HC and MC states diminish in strength, while the intensity cloud associated with the LC state becomes more prominent, as indicated by the dashed circles in Figure 1D. Additionally, we examined the stretching distance distributions of these molecular junctions as shown in the insets of Figure 1D. The plateau lengths (ΔZ) of the conductance features were measured to be about 0.56 ± 0.09 nm, 0.57 ± 0.10 nm, 0.59 ± 0.12 nm, and 0.60 ± 0.15 nm, at 10^−4^ M, 10^−5^ M, 10^−6^ M, and 10^−7^ M concentrations, respectively. Considering the gold-gold snap-back distance of 0.5 nm after the rupture of gold point contact,^32^ we determined the calibrated junction length of the molecular junction is consistent with the theoretical molecular junction length of 1.14 nm.^8^ This indicates that the LC state corresponds to a fully extended gold/4,4′-BPY/gold junction as shown in Figure 1A.

Previous studies have reported that the conductance of the 4,4′-BPY molecular junction gradually decreases as the angle between the molecule and the electrode increases.^8^ This phenomenon is attributed to the absorbed angle of the 4,4′-BPY molecule in the molecular junction, which affects the coupling between the molecule and the electrode, thus influencing the conductance. In our study, we observed a significant impact of concentration on the conductance of the 4,4′-BPY molecular junction, as well as the emergence of an additional new state (MC) alongside the well-known HC and LC states in 4,4′-BPY molecular junctions. It is likely a result of variations in the absorbed configuration of 4,4′-BPY on the gold surface caused by intermolecular interactions, which in turn affect the charge transport properties of the molecular junctions. As a result of these experimental findings, we propose plausible configurations for the three conductance states of the 4,4′-BPY molecular junction, as depicted in Figure 1A.

### Spectral clustering analysis

**Figure 2:**
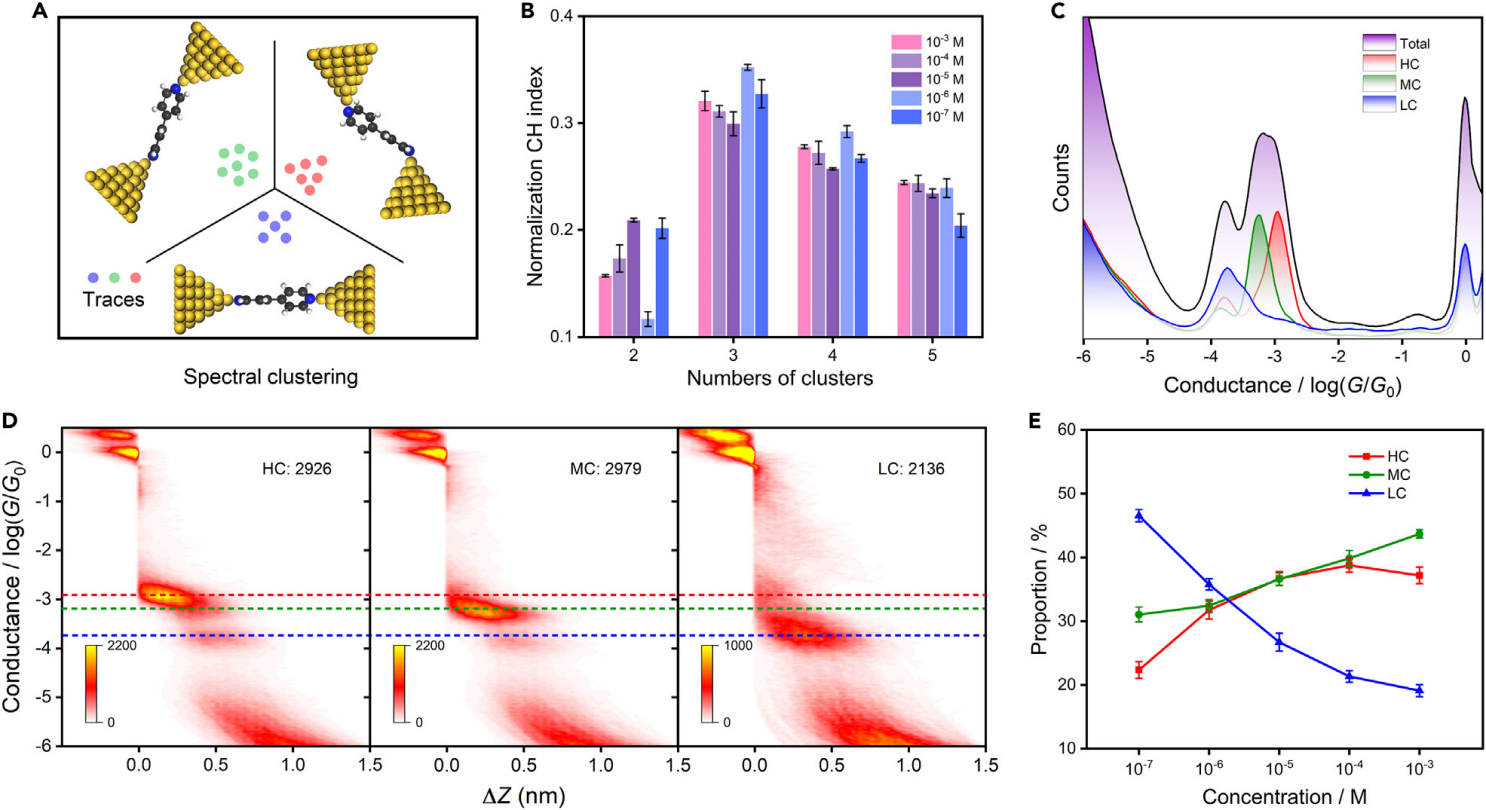
Spectral clustering results of the 4,4′-BPY molecular junction (A) Schematic diagram of conductance classification. (B) Normalized clustering results, data are represented as mean ± standard error of the mean. (C) 1D and (D) 2D conductance histograms of HC, MC, and LC after clustering at 10^−5^ M concentration. (E) The relationship between three conductance states (HC, MC, and LC) of 4,4′- BPY molecular junction and concentrations, and the Error bars were obtained by three parallel experiments.

Based on the conductance measurement results, it is challenging to distinguish between the three conductance states of the 4,4′-BPY molecular junction, particularly for the minor difference in conductance between the HC and MC. Therefore, it is necessary to utilize the clustering algorithm to classify the original conductance data. The spectral clustering method (see supplemental information for details) based on the Calinski-Harabasz (CH) proposed by Lin and Wang et al.^33^^,^^34^ can be used to divide conductance traces into multiple clusters to describe different types of conformations. Using this approach, the conductance data obtained in the experiment are classified, and the classification scheme is presented in Figure 2A. The spectral clustering method was employed to classify conductance data at all concentrations, and based on the CH index, cluster number 3 was determined to be the most appropriate for all concentrations (Figure 2B), indicating that there are three different conductance states for each of the five different concentrations.

The 1D and 2D conductance histograms after being classified at 10^−5^ M concentration are displayed in Figures 2C and 2D (classification results of other concentrations can be found in Figures S5–S8). From the 1D conductance histograms of all traces and the traces of each cluster, it can be observed that the conductance is divided into three categories, and a few LC occur in the HC and MC. Moreover, in the 2D conductance histograms after classification, a small proportion of the LC occurs in the HC and the MC as the distance between the electrodes increases, and the reason has been discussed in the previous report.^8^ This is because during the formation of molecular junctions, as the distance between the electrodes gradually increases, sometimes the molecules break directly after forming a molecular junction, and there is only one conductance platform in single conductance-distance traces. In other cases, the molecules will be slightly straightened, and multiple conductance platforms will appear in the same traces. From the classified results, it can be obtained that the HC, MC, and LC values of 4,4′-BPY molecular junctions are 10^−2.9^ *G*_0_ (97.6 nS), 10^−3.2^ *G*_0_ (48.9 nS), and 10^−3.8^ *G*_0_ (12.3 nS), respectively.

The spectral clustering method allows us to determine the number of conductance traces for each class and their ratios.^35^ We investigated the relationship between different concentrations and the proportion of three conductance states. At a concentration of 10^−7^ M, the proportions of the three conductance states are 22.37% (LC), 31.07% (MC), and 46.56% (LC), respectively, with a higher proportion of LC than HC and MC. However, at a concentration of 10^−3^ M, the proportions are 37.19% (HC), 43.70% (MC), and 19.11% (LC), respectively, with the proportion of LC much smaller than that of HC and MC. The ratio relationship of the three conductance states at all concentrations is shown in Figure 2E (the values of the proportions of HC, MC, and LC at other concentrations are shown in Table S1). We found that as the concentration of 4,4′-BPY increased, the ratio of HC and MC also increased. However, at high concentrations (10^−5^ M to 10^−3^ M), the proportion of HC does not increase appreciably but tends to stabilize. We speculate that these results likely arise from the different intermolecular interactions. Specifically, at high concentrations where the distance between molecules is relatively small, the interaction between molecules does not support an increase in the number of molecules coupled between two electrodes at small angles. Conversely, at low concentrations, molecules are more willing to couple between electrodes with an upright state due to weak intermolecular interactions. This difference in the intermolecular interactions can lead to different conductance states, resulting in a decrease in the proportion of HC and MC and an increase in the proportion of LC.

To investigate the effect of concentration on the conductance of other pyridine-linked molecular junctions, we measured the 1D and 2D conductance histograms of 1,2-bis(2-pyridyl)ethylene (BPE) in TCB at different concentrations, and the results are shown in Figure S9. Consistent with previous reports,^23^^,^^24^ we also measured that the BPE molecule junction has two distinct conductance peaks at 10^−4^ M concentration. From the 1D conductance histogram, it is observed that the peak corresponding to the HC gradually decreases with decreasing concentration. At the concentration of 10^−6^ M, a shoulder peak appears, while the HC peak is not distinct and it becomes difficult to distinguish between the MC and LC. At the concentration of 10^−7^ M, only a small LC peak appears in Figure S9. By applying the spectral clustering method on the BPE conductance data of all the concentrations (10^−4^ M to 10^−7^ M), we obtained the relationship between the concentration and the proportions of three states in the BPE molecular junctions (Figure S10), and similar results were also obtained, which indicates that these effects are not specific to the 4,4′-BPY molecular junctions.

As additional controls, we measured the conductance of 4,4′-BPY in the volatile mix solvent consisting of 1,3,5-trimethylbenzene and tetrahydrofuran [V(TMB): V(THF) = 4:1] to verify the effect of solvent evaporation rate on conductance measurement at 10^−4^ M concentration, as shown in Figure S11. It can be seen that over time, the conductance peak of the 4,4′-BPY molecular junction hardly changes until the mixed solvent is completely volatilized (the mixed solvent is almost volatilized at 70 min). These experiments further exclude the effect of solvent/molecular environment on the conductance of molecular junctions. In summary, we can therefore conclude that the conductance changes with the concentration in the molecular junction are mainly caused by the influence of the number of molecules (intermolecular interactions), all of which reinforced our hypothesis. Finally, we must emphasize the importance of the excellent differences observed in these single-molecule measurements in this study, which makes it possible to investigate the effects of intermolecular interactions on charge transport properties. STM-BJ data provide details of molecular interactions that are difficult to achieve through other experimental techniques.^6^^,^^12^^,^^25^

### Flicker noise analysis

**Figure 3:**
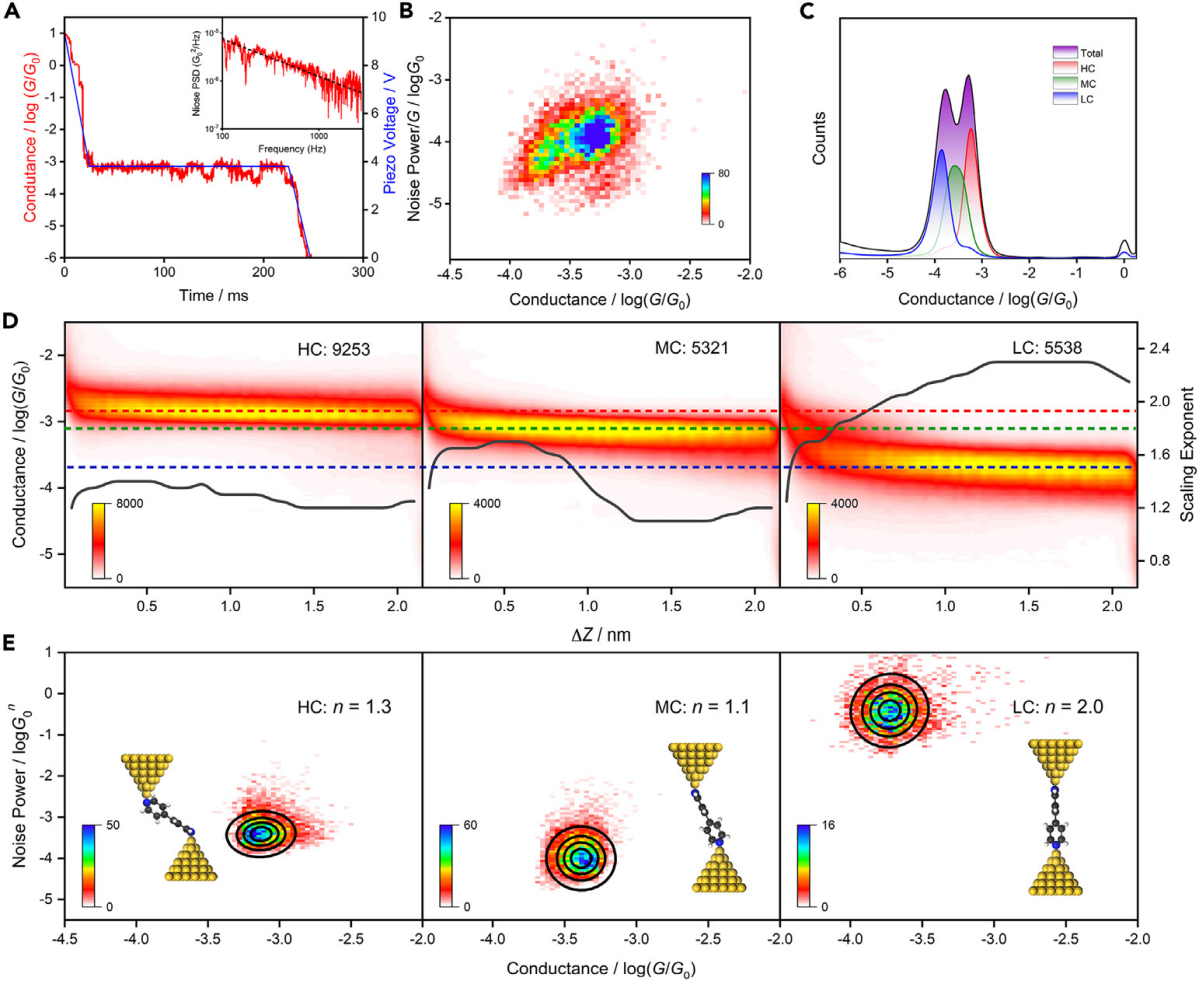
Analysis results of flicker noise (A) The red line is the typical single conductance trace in hover mode, and the blue line represents the piezo voltage during the break junction process, inset: the relationship between PSD and frequency. (B) The normalized 2D histogram of the relationship between PSD and conductance for 4,4′-BPY at 10^−6^ M concentration (21,041 traces). (C) The 1D hovering conductance histograms of HC (7,571 traces), MC (7,541 traces), and LC (5,929 traces) after clustering at 10^−4^ M concentration. (D) The exponent curve (black solid line) and 2D hover conductance histograms of HC, MC, and LC of the 4,4′-BPY molecular junction after clustering at 10^−4^ M. (E) The normalized 2D histogram of the relationship between PSD and conductance for HC, MC, and LC after clustering at 10^−6^ M concentration. Dotted contours represent fits to the bivariate normal distribution. The insets in (E) show its possible molecular junction configurations. The parameter *n* represents the noise power scaling exponents.

The flicker noise in single-molecule junctions varies with the coupling between molecular and electrode, which is caused by the switching between metastable configurations of molecule junctions. Previous research has demonstrated that flicker noise is power-law dependent on conductance, which can be used to determine the charge transport mechanisms (through-bond and through-space) of the electrode and molecule.^27^^,^^36^^,^^37^ Specifically, when the dependency exponent approaches 1.0, it indicates a through-bond transport, whereas an exponent near 2.0 signifies a through-space transport mechanism (details described in supplemental information). We investigated the flicker noise in single-molecule junctions of 4,4′-BPY at the concentration of 10^−4^ M, 10^−5^ M, and 10^−6^ M under the hover mode. The 1D hovering conductance histogram for all concentrations is shown in Figure S12, and there are two obvious conductance peaks in all concentrations, consistent with the results obtained during normal measurement (Figure 1C). The representative single-conductance trace in hover mode and the relationship between noise power spectral density (PSD) and frequency trajectories are shown in Figure 3A. The PSD is obtained by Fourier transform and squares the conductance data in hover mode. From the illustration, it can be seen that in the frequency range of 100–1000 Hz, the PSD presents flicker noise (1/*f* noise) characteristics, which is similar to the ref.^27^ Moreover, the normalized 2D histogram of the relationship between PSD and conductance is shown in Figure 3B. It can be found that there are three obvious modules in the normalized 2D histogram (the other two concentrations are shown in Figure S13).

To distinguish the charge transmission modes of these states, the hovering conductance data are separated by the spectral clustering algorithm. The 1D hovering conductance histograms after clustering at 10^−4^ M concentration are shown in Figure 3C (other clustering results are shown in Figure S14). As expected, there are three different conductance states obtained after clustering, which is consistent with the clustering results in the previous section. Our previous work^37^ highlights the use of time-frequency analysis to investigate the relationship between flicker noise and conductance during the dynamic breaking process of molecular junctions, providing insights into the charge transport mechanism in single-molecule junctions. We further used this method to analyze the flicker noise of the conductance data classified into three different concentrations mentioned earlier (As shown in Figures S15 and S16). Specifically, we focused on the 10^−4^ M concentration for a detailed explanation, as depicted in Figure 3D. The solid black line represents the variation of the *n* value. The observed *n* values do not strictly adhere to 1.0 or 2.0, but rather exhibit fluctuations between these values. This suggests that the transport mechanism in this system is not solely determined by through-bond or through-space interactions. Specifically, both π-binding and σ-binding are involved in charge transport in all three conductance states. Interestingly, we observed only a slight change in the *n* around 1.3 for HC, whereas MC and LC showed larger fluctuations (compared to HC) with the scaling exponent tending to stabilize in the latter half of hovering. These observations can be attributed to the tilted molecular configuration between the two electrodes in different conductance states. In HC, the π-binding and σ-binding collectively influence charge transfer, resulting in a fluctuation of the value of *n* around 1.3. As the distance between the electrodes increases, the molecular configuration changes and the *n* value becomes greater than 1.3. Subsequently, the contribution of Au-N bonds increases, leading to a decrease in the value of *n*. Once the configuration stabilizes, *n* tends to reach a steady state. Ultimately, in LC, the molecule stands upright, and the distance between the electrodes causes the contribution of the Au-N bond to gradually weaken. This leads to an increase in the value of *n* within the system until the Au-N bond eventually breaks. This aligns with the recent perspective put forward by Baloch et al.^38^

Subsequently, PSD analysis was performed on the three clustering conductance data, and the results are shown in Figure 3E (details shown in Figures S17–S19). By plotting the normalized 2D histogram of the relationship between PSD and conductance, we find that the noise power scaling exponents *n* for HC at three concentrations were 1.4 (10^−4^ M), 1.3 (10^−5^ M), and 1.3 (10^−6^ M), respectively. Similarly, the *n* values of MC are 1.2, 1.1, and 1.1, respectively. This is the result of the collective influence of π-binding and σ-binding. However, different from HC and MC, the values of *n* in LC are 2.0, 1.7, and 2.0, respectively. These results align with the findings presented in Figure 3D, which indicates the strong coupling between molecules and electrodes in the HC and MC, whereas the coupling in the LC is relatively weak. It further verifies that the configurations of the three conductance states of the 4,4′-BPY molecular junction proposed in Figure 1A are reasonable. These results are particularly important for the following theoretical calculations and discussions.

### DFT calculations

**Figure 4:**
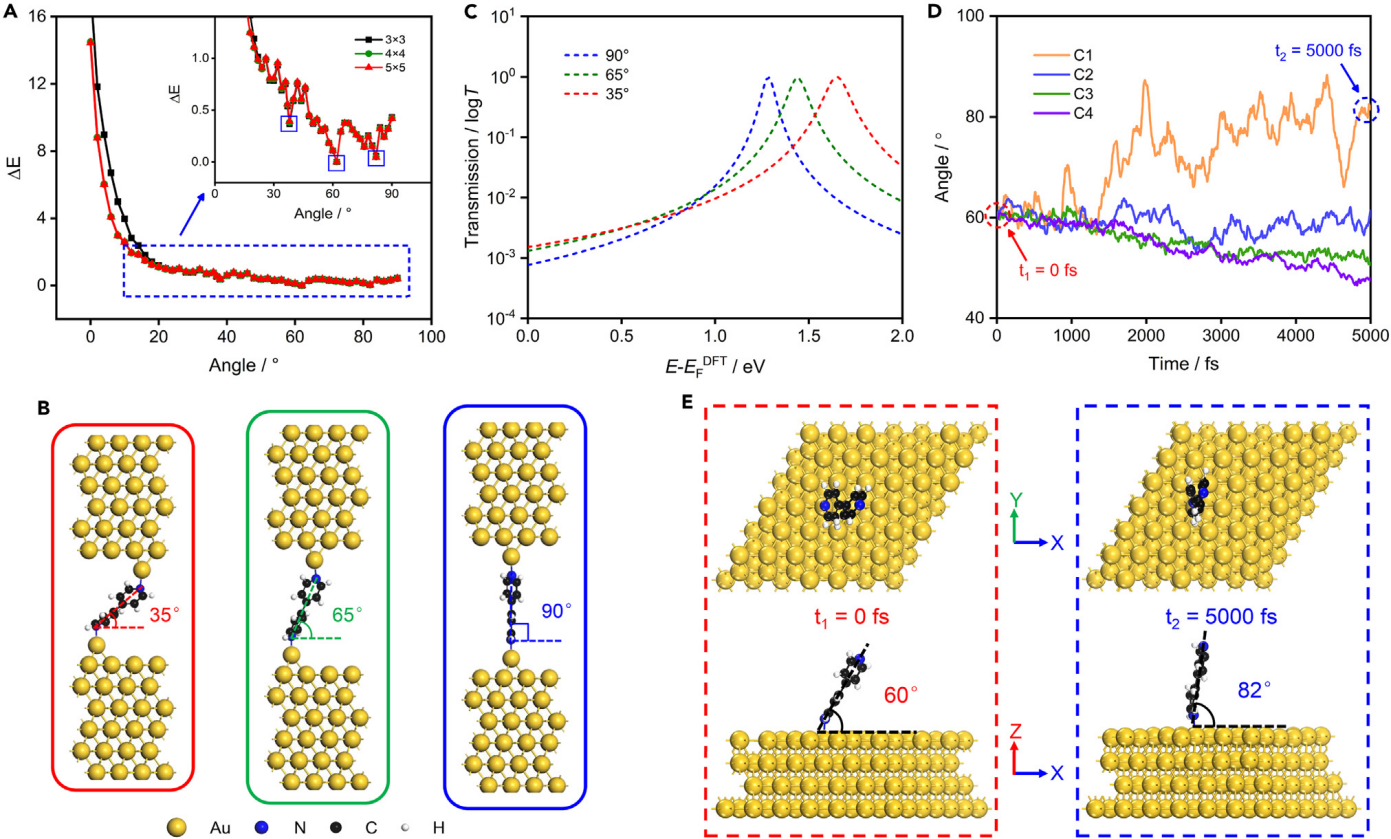
Theoretical calculation results (A) The relationship between total energies and angles, the corresponding total energies are plotted relative to the lowest energy value (the number in the upper right corner of the insert shows the size of the Au(111) supercell). The angle is defined as between the line between two nitrogen atoms and the Au(111) surface. (B) The configuration of the three conductance states of 4,4′-BPY molecular junctions after DFT optimization, where HC, MC, and LC are from left to right. (C) Self-energy corrected transmission functions of the three conductance states, the red, green, and blue dotted line represents 35°, 65°, and 90°, respectively. (D) Angle of 4,4′-BPY relative to Au(111) surface during AIMD simulation for C1 to C4 (the C1 represents that there is one 4,4′-BPY molecule on the surface of 6 × 6 Au(111) supercell, C2 represents that there are two 4,4′-BPY molecules on the surface, and so on). (E) Top and side view of the structure of initial (left) and after 5000 fs (right) in AMID simulation for C1.

To further clarify the configuration corresponding to different conductance and the effect of molecular concentration on the configuration of the molecular junction, we carried out the first-principles calculations by using the Vienna ab initio simulation package (VASP)^39^^,^^40^ (details described in supplemental information). The most stable configuration of the 4,4′-BPY adsorbed on the Au(111) surface is that the N atom is located at the top of the Au atom, which is in agreement with the previous studies.^41^^,^^42^ Based on this, the energy of different angles has been considered while the molecule is adsorbed on the top of the Au(111) surface (Figure S20). The results indicated that there are three obvious energy valleys corresponding angles are about 38°, 62°, and 82° (see Figure 4A), respectively (the angle is defined as between the line between two nitrogen atoms and the Au(111) surface). Therefore, we use three relatively stable angles of 4,4′-BPY adsorption on the Au surface as the initial angle of the 4,4′-BPY molecular junction for structural optimization. After full optimization, three stable structures with dihedral angles of about 35°, 65°, and 90° are obtained, as shown in Figure 4B. It is verified that the 4,4′-BPY molecular junction has three conductance configurations.

Moreover, the transmission functions are calculated using Quantum ATK.^43^ The transmission spectra of these three configurations are calculated using self-energy correction and Lorentz fitting^44^^,^^45^ (details described in supplemental information). Previous calculations^8^^,^^44^^,^^45^ indicate that the essential channel supporting charge transport in 4,4′-BPY is the lowest unoccupied molecular orbital (LUMO). Therefore, the transmission functions on the right side of the Fermi level (*E*_F_) for three configurations are given, which shows that the width of the LUMO-derived transmission peak increases with the increase of angle, as shown in Figure 4C. It is worth noting that the transmission peak width derived from LUMO decreases with the increase of angle, which indicates that the smaller the angle is, the stronger the coupling strength between molecules and electrodes is. Then, the calculated binding energies of the three configurations, are 0.59 eV (35°), 1.11 eV (65°), and 1.67 eV (90°), respectively, which is consistent with the trend in the previous report.^8^ It can be found that the coupling between molecule and electrode changes from π to δ with the increase of angle, and the coupling becomes weak, resulting in the increase of binding energy. Moreover, the transmission eigenstates of the molecular junction also verify the relationship between angle and coupling, as shown in Figure S22. It shows that the larger the molecular angle between the electrodes, the weaker the coupling between them, and the smaller the conductance of the molecular junction. The results from transmission spectra and binding energies are consistent with the conductance trend observed in the experiment.

We further simulate the angle changes of 4,4′-BPY on the Au(111) surface with different concentrations by means of the AIMD method. Here, the different numbers of 4,4′-BPY molecules placed on the same size Au (111) surface to represent different concentrations, and four different concentrations C1, C2, C3, and C4 are considered, as shown in Figure 4D. In the initial structure, the angle between the molecule and Au(111) in all concentrations is 60°, while after 5000 fs in AIMD simulation, the angles for C1, C2, C3, and C4 are 82.2°, 61.4°, 50.9°, and 47.6°, respectively. The structure of initial and after 5000 fs in AIMD simulation for C1 is shown in Figure 4E (other structures are shown in Figures S23–S25). Notably, it can be found that the higher the concentrations, the smaller the angles, when the concentration is lower, it is closer to being upright. This is consistent with our conjecture that molecules are more willing to adsorb on the electrode with an upright state at low concentrations. Simultaneously, it indicates that the greater the interaction between molecules, the smaller the angle of molecule adsorption on the Au(111) surface, which will result in the stronger ability of charge transport with the molecular junction. This is the main reason why the concentration affects the charge transport properties of the 4,4′-BPY molecular junctions. These findings provide insights into the effect of intermolecular interactions on the charge transport properties of molecular junctions, which is of great significance for designing and optimizing molecular electronic devices.

### Conclusion

In summary, we employed an STM-BJ technique to investigate the single-molecule absorption configuration between the pyridine-terminated molecules and the Au electrode surface at varying concentrations ranging from 10^−7^ M to 10^−3^ M. Our results indicate the 4,4′-BPY molecular junctions exhibit an intermediate conductance (MC) state in addition to the conventional high (HC) and LC states. To quantitatively analyze the distribution of each conductance state with respect to concentration, we applied a spectral clustering algorithm, revealing a significant dependence on concentration among the three conductance states. Additionally, through flicker noise analysis, the injection of charges from the electrode into the molecule was found to differ in both through-bond and through-space pathways among these three conductance states. The theoretical calculations reveal that the concentration modifies the adsorbed molecule geometry of the molecular junctions on the Au electrode surface, thereby influencing charge transport within the system. Our research introduces a straightforward and achievable method for controlling adsorption configurations in single-molecule junctions, thus promoting the advancement of future molecular devices.

### Limitations of the study

In this study, we observed three conductance states that align with the results of theoretical calculations. However, we do not have more direct experimental evidence to indicate that the actual adsorption configuration on gold electrode corresponds to these three angles, although flicker noise can provide evidence to a certain extent.

## STAR★Methods

### Key resources table

**Table:** 

REAGENT or RESOURCE	SOURCE	IDENTIFIER
**Chemicals, peptides, and recombinant proteins**		

4,4'-Bipyridine	Aladdin	CAS: 553-26-4
1,2,4-Trichlorobenzene	Aladdin	CAS:120-82-1
1,2-bis(2-pyridyl)ethylene	Aladdin	CAS: 13362-78-2
1,3,5-Trimethylbenzene	Aladdin	CAS: 108-67-8
Tetrahydrofuran	Aladdin	CAS:109-99-9

**Software and algorithms**		

Quantum ATK	Synopsys Inc.	https://www.synopsys.com/
VASP	VASP Software GmbH	https://www.vasp.at/
Python 3.12	Python Software Foundation	https://www.python.org
VESTA software package	JP-Minerals	https://jp-minerals.org/vesta/en/

### Resource availability

#### Lead contact

Further information and requests for resources and reagents should be directed to and will be fulfilled by the lead contact, Yong Hu (yhu@xtu.edu.cn).

#### Materials availability

This study did not generate new unique reagents.

#### Data and code availability

The simulation data and code that support the findings of this study are available from the lead contact upon reasonable request.

Any additional information required to reanalyze the data reported in this paper is available from the lead contact upon request.

### Experimental model and study participant details

#### Experimental

Preliminary preparation: Gold wire (99.99%, 0.25mm in diameter) was purchased from Beijing Jiaming Platinum Nonferrous Metal Co, Ltd. for making STM tips. To make the gold bead, clean the gold tip with a flame. For the substrate: first clean the silicon wafer (<100>single crystal), heat the solution [V (H_2_O): V (HCl): V (H_2_O_2_)= 5:1:1] to 35°C, put the silicon wafer in the solution and soak it for 15 minutes, then take it out clean with ultra-pure water (produced by Milli-Q system with a resistance of 18 MΩ.cm), and dry it with nitrogen. Then, 10 nm titanium film and 200 nm gold film were deposited on silicon wafer by DE500 magnetron sputtering deposition system from Beijing DE Technology Limited Co, Ltd. In this study, we used 1,2,4-trichlorobenzene (TCB) as a solvent to prepare 4,4'-bipyridine (4,4'-BPY) solutions with different concentrations, TCB and 4,4'-BPY were purchased from Aladdin.

#### Theoretical calculations

First-principles calculations were performed in the Vienna ab initio simulation package (VASP)^40^ based on density functional theory (DFT), and the generalized gradient approximation (GGA) of the Perdew–Burke–Ernzerhof (PBE)^46^ are described as the exchange-correlation energy. The energy cutoff and the k-points are set to be 500 eV and 2×2×1 for optimization structures, respectively. The convergence of energy and force is set to be 10^-6^ eV and 0.01 eV/Å, respectively. To avoid the interlayer interaction periodic boundary conditions, the vacuum spacing is set to be 20 Å between adjacent layers. Semiempirical DFT-D3 correction was performed to describe the van der Waals interactions. To calculate the transmission function, the molecules are located in the middle of gold electrodes connecting with a single atom. In the calculation of Quantum ATK, the single-ς polarization basis is set for the gold element and double-ς for other elements, and the real space grid is defined by the equivalent energy cut-off of 75 Hartree. The k-points of 3×3×50 were used for transfer function calculation. The transmission eigenstates corresponding to the Fermi level are derived, and its isovalue is 0.2.

#### Geometric optimization

The model of three four-layer Au(111) surfaces, including 3×3, 4×4 and 5×5 Au(111) supercells, were adopted in our work. The nitrogen atom of the 4,4'-BPY molecule is located at the top of the gold atom with a distance of 2.2 Å. The energies of the molecular angle in this structure from 0° to 90° were calculated, and the results are shown in Figure 4A. The structural diagram taking 4×4 as an example is shown in Figure S20. The binding energy of the two monomers was calculated by the equilibrium method,^47^ using the following equation:

$$E_{b}=E^{\text{AB}}\;-\;E^{A}\;-\;E^{B}$$

Where $E^{\text{AB}}$ is the total energy, $E^{A}$ and $E^{B}$ are the energies of two monomers respectively.

#### Ab initio molecular dynamics simulation

Ab initio molecular dynamics (AIMD) simulations of C1 to C4 with the canonical (NVT) ensembles were performed at room temperature (300K). Here, the Cn represents that there are n 4,4'-BPY molecules on the 6×6 supercells Au(111) surface. The top view and side view of the initial (left) and after 5000 fs (right) structures in AIMD simulation for C2 to C4 are shown in Figures S23–S25 (a), and the initial angle between the molecule and surface in all structures are set to be 60° (the angles mentioned in this part are the angles between the line of two nitrogen atoms of the 4,4'-BPY molecule and the Au surface). The change of angles is shown in Figures S23–S25 (b) during AIMD simulations, where the gray lines and red lines represent the angle of each molecule and the average angle, respectively. After AIMD simulations, the average angles between the molecule and Au(111) surface are about 61.4°, 50.9°, and 47.6° for C2, C3, and C4 structures, respectively. Clearly, the angle decrease with the increase of 4,4'-BPY molecules, which indicates that the concentration of molecular influence the angle between the 4,4'-BPY molecules and the Au(111) surface.

### Method details

#### Spectral clustering algorithm

The spectral clustering algorithm is a clustering method that segments data and divides similar samples into a cluster. Based on Lin and Wang et al.,^33^^,^^34^ given the conductance histogram data *H* = {*h*_*1*_*, h*_*2*_*,..., h*_*M*_} in ***R***^*N*^ (divide the conductance axis into discrete N bins), and cluster it into *K* clusters: (1) Form the affinity matrix ***A***∈***R***^*M×M*^ defined by ***A***_*ij*_ *=* ***C***_*ij*_+1, if *i ≠ j*, and ***A***_*ij*_ = 0. (2) Define ***D*** to be the diagonal matrix whose (*i, j*)-element is the sum of ***A***’s ith row, and construct the matrix ***L*** =***D***^−1/2^***AD***^−1/2^ – ***I*** (***I*** is a unit matrix). (3) Find *x*_1_, *x*_2_, ... *x*_K_, the *K* largest eigenvectors of ***L***, and form the matrix ***X*** = [*x*_1_, *x*_2_, ... *x*_K_] belonging to ***R***^*M×K*^ by stacking the eigenvectors in columns. (4) Treating each row of ***X*** as a point in ***R***^*K*^, cluster them into *K* clusters via *K*-means++. (5) If and only if row *i* of the matrix ***X*** was assigned to cluster *j*, the original points *h*_*i*_ to cluster *j*. In this study, the methods and steps we used are almost the same as those described by Lin and Wang et al..^33^^,^^34^ ***C***_*ij*_ is defined as the correlation between histogram *h*_*i*_ and *h*_*j*_, as follows:

$$C_{ij}=\frac{\left\langle \left[h_{i}-\left\langle h_{i}\right\rangle \right]\left[h_{j}-\left\langle h_{j}\right\rangle \right]\right\rangle }{\sqrt{\left\langle {\left[h_{i}-\left\langle h_{i}\right\rangle \right]}^{2}{\left[h_{j}-\left\langle h_{j}\right\rangle \right]}^{2}\right\rangle }}$$

where ⟨*h*_*i*_⟩ is the average value of histogram *h*_*i*_, and the value range of ***C*** is [-1, 1]. To meet the requirements of spectral clustering, we add elements that make the affinity matrix ***A*** non-negative. *h*_*i*_ is the conductance histogram for the *i*-th individual trace, *M* is the number of conductance traces, and *N* is the number of the histogram bins. Moreover, the CH index is a promising index for evaluating the number of clusters *K*. The maximum index value represents the optimal number of clusters in the data set.

It combines the Calinski - Harabasz (CH) index as the standard, to divide the obtained conductance traces into different clusters, which can be used as different types of junction conformation events that would occur during the single-molecule break junction process. First, the original conductance data obtained from the experimental measurements are clustered; Multiple classification cases can be obtained by using the program (we have considered two to five). Then, the CH index is used to judge the optimal classification (the maximum of the index represents the optimal number of clusters). The CH index shows that the optimal classification of all five concentrations is 3 (Figure 2B). The 1D and 2D conductance histograms of the other four concentrations classified by the clustering algorithm are shown in Figures S5–S8.

#### Power Spectral Density (PSD)

STM-BJ technique can repeatedly construct a large number of molecular junctions in a short time, to obtain a large number of hovering curves of single-molecule junctions for statistical analysis of noise power spectral density (PSD). For flicker noise analysis, during the measurement, paused for 200 ms at the position where the molecular junction may form, as described in the previous work.^27^ The noise power spectral density is obtained by performing Fourier transform and square transformation on the hovering conductance platform. Next, the PSD of 100 to 1000 Hz is integrated to obtain the quantized PSD of each trace, and the integrated PSD is normalized by the average conductance of the cut-out traces (the conductance range is 10^-2.5^ G_0_ to 10^-4.5^ G_0_). Additionally, to determine the conductance PSD of the junction, the conductance measured during the fixed displacement section of the junction was analyzed. We draw the contour line according to the fitted Gaussian distribution equation. Then, we increase the noise power of *G*^*n*^ from *G*^1.0^ to *G*^2.0^ in steps of 0.1 to analyze whether the electrode-molecule coupling is through-bond or through-space.

The flicker noise power of the curve can be obtained by integrating the frequency range of 100 ∼ 1000 Hz, The formula is:

$$\delta \left[x\left(t\right)\right]=\int_{100}^{1000}S_{x}\left(f\right)df$$

Then calculate the conductance value *G* when hovering. Count the ratio of flicker noise power to the average conductance value of all hovering curves: δ[*x(t)*]/ *G*, and then make a 2D histogram based on the ratio and conductance value *G*. and finally, an exponent *n* is obtained by:

$$n=\text{argmi}n_{n}\left|\text{corrcoef}\left(G,\frac{\delta \left[x\left(t\right)\right]}{G}\right)\right|$$

where corrcoef (a,b) is a function calculating the Pearson correlation coefficient of variables a and b. A key point here is to ensure the accuracy of the estimated flicker noise. Since the noise power is obtained by integrating the square of the amplitude spectrum of conductance trace after the discrete Fourier transforms, the result of the Fourier transform must be accurate first. For a specific frequency component, sufficient cycles of periods should be taken to ensure accuracy. We have selected 20112, 20664, and 21041 conductance curve traces respectively under hover modes with three different concentrations of 10^-4^ M, 10^-5^ M, and 10^-6^ M.

To further investigate the charge transfer mechanism under different conductance states, the classified hover data under different concentrations (10^-4^ M, 10^-5^ M, and 10^-6^ M) were analyzed for continuous Flicker noise using Python programming. The calculation method is the same as the above Flicker noise analysis. However, instead of noise analysis on the conductance data over the entire hovering range and obtaining the minimum scaling exponent, we use a sliding window to divide the conductance data within the hovering range into many segments, as shown in Figure S15 (a). For calculation convenience, a window width of 1000 points was used (the black box in Figure S15 (a)), and the window sliding with a stride s = 100 points on the selected range of the junction period, and then interpolate back to the same length as the selected range. Then conduct a Flicker noise analysis on the conductance data in the fixed window to obtain the minimum scaling exponent in the window, as shown in Figure S15 (b). With the sliding of the window, the relationship between the scaling exponent and hover time can be obtained (black line in Figure S16).

#### Transmission function calculation

DFT calculation enables the relatively efficient exploration of the relationship between structure and conductance. But the resonance position E_R_ is too small due to the inherent self-energy error of using (LDA or) GGA Kohn-Sham (KS) eigenvalue as quasiparticle energies. However, to obtain more accurate conductance estimation, it is necessary to carry out a self-energy correction at the molecular level. We refer to the methods in References^8^^,^^24^^,^^44^^,^^45^ for self-energy correction Σ of the calculated transmission function.

The estimation of Σ requires two steps: (1) correcting the Kohn Sham LUMO eigenvalue (previous studies found that the correction of HOMO resonance did not significantly change the transmission coefficient near Fermi energy (*E*_*F*_), so we only focused on LUMO resonance) in a gas-phase (the value is expressed in $\Sigma_{0}^{\text{LUMO}}$). For the gas phase 4,4'-BPY molecule, the comparison between the measured ionization potential^48^ and the eigenvalue value of GGA LUMO (relative to vacuum) implies a self-energy correction of $\Sigma_{0}^{\text{LUMO}}$ ∼3.01 eV. (2) Using an image charge model considering electrode polarization in junction to further correction of gas-phase correction (the value is expressed in W). The model assumes that the electrode is a perfect conductor and contains an infinite series of images on both sides of the junction. To estimate W, we express the electrons in LUMO as point charges between two image planes and take 1Å from the Au (111) surface on both sides of the junction. In this instance, the value of *W* = (*e*^2^ln2) / (8πε_0_*a* ) (eV), where *a* is the distance between the point charge and the image planes, the values are shown in Table S2. The specific value of Σ is the final Σ^LUMO^ relative to *E*_*F*_ (eV), the $\Sigma^{\text{LUMO}}$ = $\Sigma_{0}^{\text{LUMO}}-W$.

The Σ-corrected conductance *G* by Lorentzian fit, the formula is as follows:^8^^,^^24^^,^^49^

$$T\left(E\right)=\frac{\Gamma_{1}\Gamma_{2}}{{\left(E\;-{\;(E}_{R}+\Sigma )\right)}^{2}+{\left(\frac{\Gamma_{1}{+\Gamma }_{2}}{2}\right)}^{2}}$$

Where $E_{R}$ is the resonance peak position, $\Gamma_{1}$ and $\Gamma_{2}$ was the self-energies describe the contact between the molecule and left and right electrodes.

As shown in Figure S22, we calculated the transmission eigenstates of the 4,4'-BPY molecule junction at the *E*_*F*_ position (after correction). For 35°, 65°, and 90°, the energy positions before correction are -1.35 eV, -1.17 eV, and -1.03 eV respectively. According to Figure S22 (a), the coupling between electrode and molecule is mostly the δ coupling between Au and N atom. (b) and (c) shows that the coupling is π coupling between Au and pyridine ring, and the coupling of 35° is more obvious. This phenomenon further illustrates that the molecular angle affects the conductance of the molecular junction, and the smaller the angle, the greater the conductance.

### Quantification and statistical analysis

During the measurement process, the migration of metal electrode atoms and the variation of adsorption sites on the metal surface by molecules can affect the measurement values, leading to certain deviations. Therefore, we need to conduct a large number of experiments, repeated multiple times, to obtain a significant amount of data in order to average out the deviations and obtain the most reliable electrical properties information. At the process of break junction, the tunneling current between the electrodes exponentially decays with the distance between the electrodes after the electrodes are opened. Taking the logarithm of conductance values with a base of 10, the conductance values exhibit a linear relationship with the distance between the electrodes.

In order to further analyze the experimental data, one-dimensional (1D) and two-dimensional (2D) statistics were performed. A section ranging from 10^-9^ *G*_0_ to 10^1^ *G*_0_ of the recorded data during the break process was extracted. Within this range, an interval of 0.01 was chosen, and the entire section was divided into 1000 intervals. The data points of the conductance-displacement curve will fall into these intervals. For each evenly divided interval, a count of 1 was assigned if a data point fell within the interval. Finally, the 1D conductance histogram was plotted with the conductance intervals as the x-axis and the number of data points as the y-axis, allowing the peak value in the chart to determine the interval with the highest probability of conductance distribution, thus determining the conductance of a single molecular junction. The peak value was obtained through Gaussian fitting.

The 2D conductance histogram is a result of statistical analysis between the conductance values and the distance values between the electrodes. Firstly, the conductance and the distance dimension were divided into regions to form rectangular areas. The range from -0.5 nm to 2.5 nm for the distance was divided into 500 equal intervals, while the conductance range from 10^-9^ *G*_0_ to 10^1^ *G*_0_ was divided into 1000 equal intervals. Then, each conductance-distance curve was traversed across all rectangular areas, and the number of points in each rectangular area was counted. Finally, the statistical results of all conductance-distance curves were accumulated to obtain the 2D conductance-distance histogram.

In cases where there are clear conductance clouds in the 2D conductance-distance histogram (such as Figure S3B), the stretching distance statistics are performed (ranging from 10^-4.5^ *G*_0_ to 10^-0,3^ *G*_0_), and the results are further subjected to Gaussian fitting. Considering the gold-gold snap-back distance of 0.5 nm after the rupture of gold point contact,^32^ the actual stretching distance is the maximum value after fitting, plus a correction of 0.5 nm.
