# Supplementary material for: Tuning charge transport by manipulating concentration dependent single-molecule absorption configurations
Source: iScience. 2024 Feb 20;27(3):109292. doi: 10.1016/j.isci.2024.109292 (PMC10910293; doi:10.1016/j.isci.2024.109292)
Supplement: Document S1. Figures S1–S25 and Tables S1 and S2 [file mmc1.pdf]

iScience, Volume *27*

Supplemental information

Tuning charge transport by manipulating concentration dependent single-molecule absorption configurations

Xia Long, Wangping Xu, Tingting Duan, Liyan Lin, Yandong Guo, Xiaohong Yan, Juexian Cao, and Yong Hu

Supplemental Information

Concentration-dependent configuration changes in single-molecule dipyridyl junctions

Xia Long,[1] Wangping Xu,[1] Tingting Duan,[1] Liyan Lin,[2] Yandong Guo,[2] Xiaohong Yan,[2] Juexian Cao[1,*] and Yong Hu[1,*]

[1]Hunan Institute of Advanced Sensing and Information Technology, Xiangtan University, Xiangtan, China

[2]College of Electronic and Optical Engineering, Nanjing University of Posts and Telecommunications, Nanjing, China

*Correspondence: jxcao@xtu.edu.cn and yhu@xtu.edu.cn

Lead contact: yhu@xtu.edu.cn

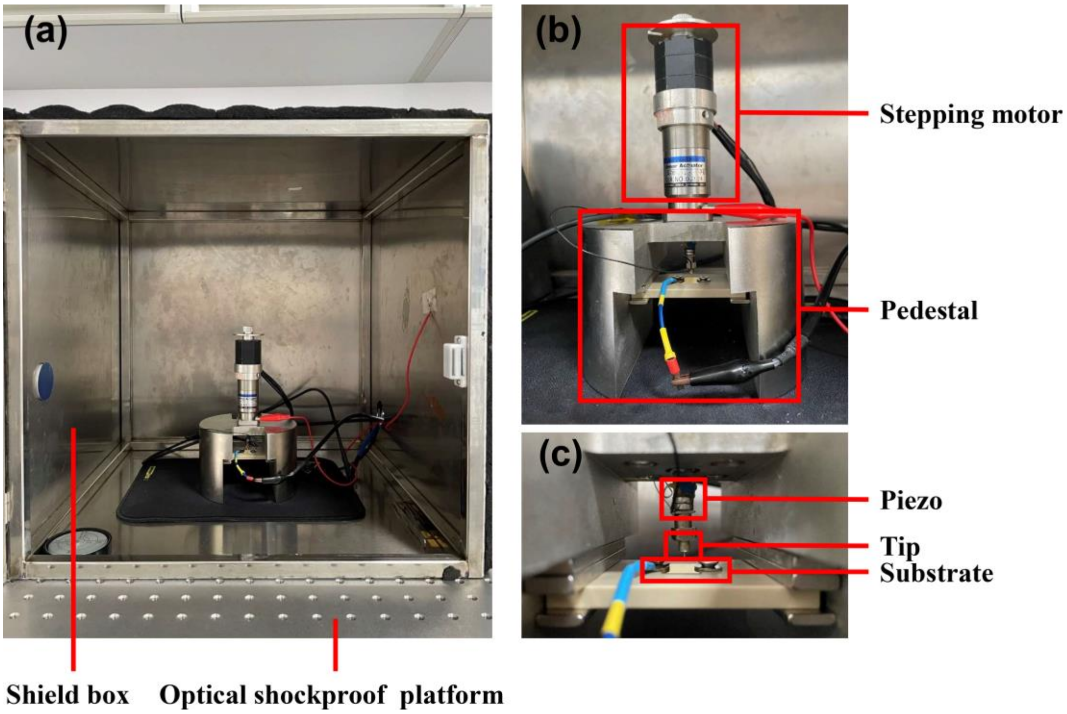

Figure S1. STM-BJ detail display diagram, related to the STAR Methods. (a) STM experimental operations section. (b) The pedestal partm and (c) the Au tip and substrate of the experimental equipment.

The conductance histograms of molecular junctions

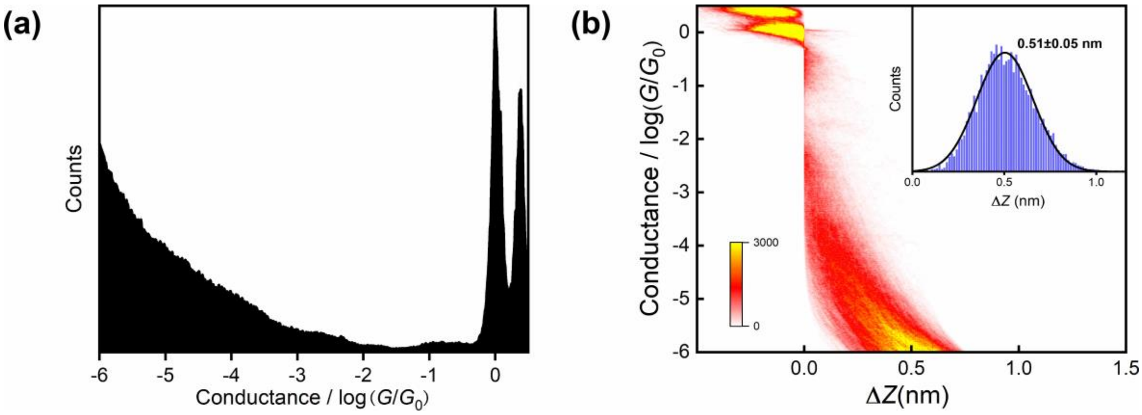

Figure S2. The conductance histograms of the pure solvent of TCB, related to Figure 1. The 1D (a) and 2D (b) onductance histogram of TCB and stretched distance distributions ranging from $10^{-0.3}$ G_0 to $10^{-6.0}$ G_0 (Insert).

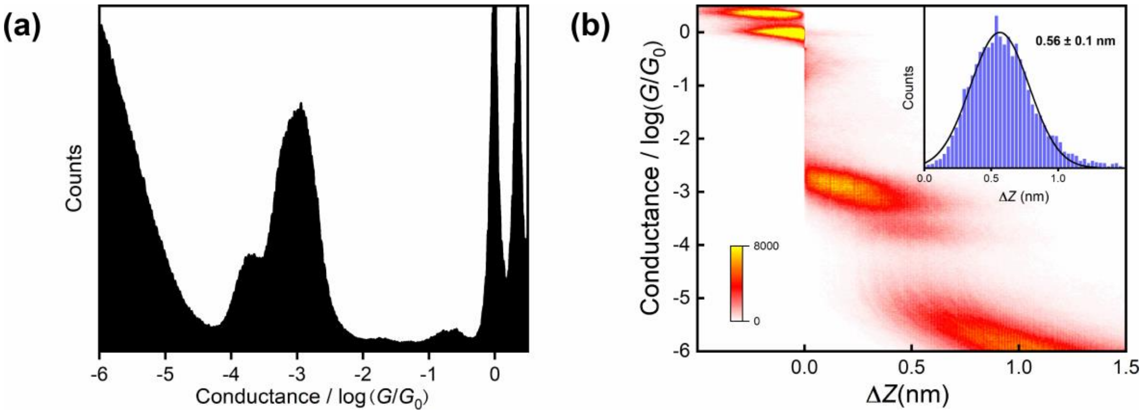

Figure S3. The conductance histograms for 4,4'-BPY in TCB, related to Figure 1. The 1D (a) and 2D (b) conductance histogram of 10^{-3} M (7,425 traces), and stretched distance distributions ranging from $10^{-0.3}$ G_0 to $10^{-4.5}$ G_0 (Insert). Conductance measurements were carried out at room temperature in a solution.

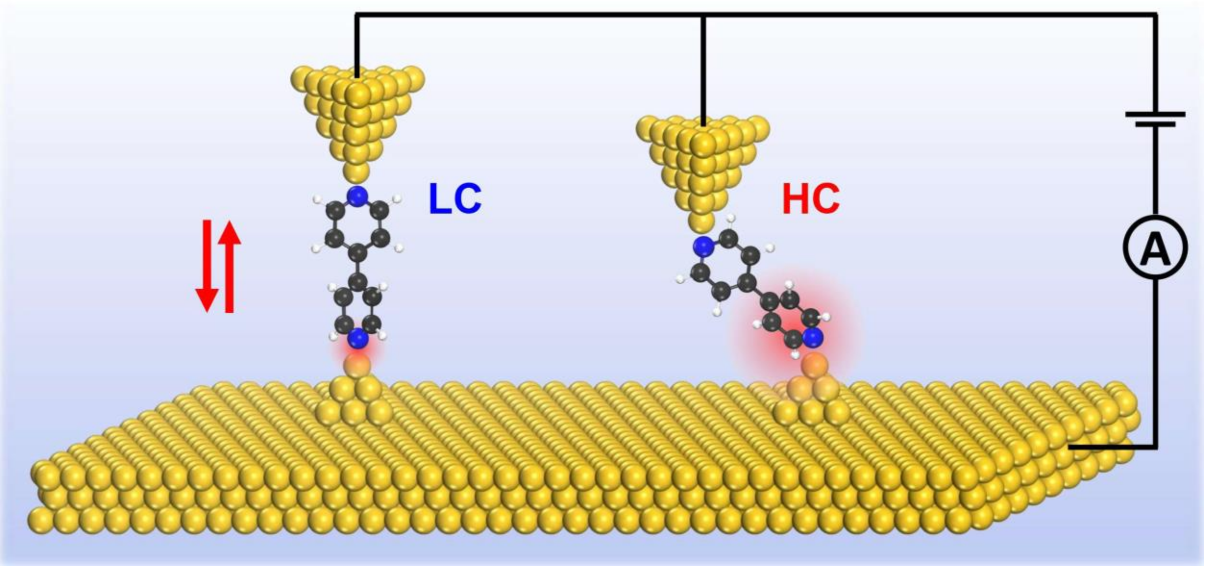

Figure S4. STM-BJ schematic diagram of 4,4'-BPY molecular junctions, related to Figure 1. The configuration of HC and LC of 4,4'-BPY molecular junctions.

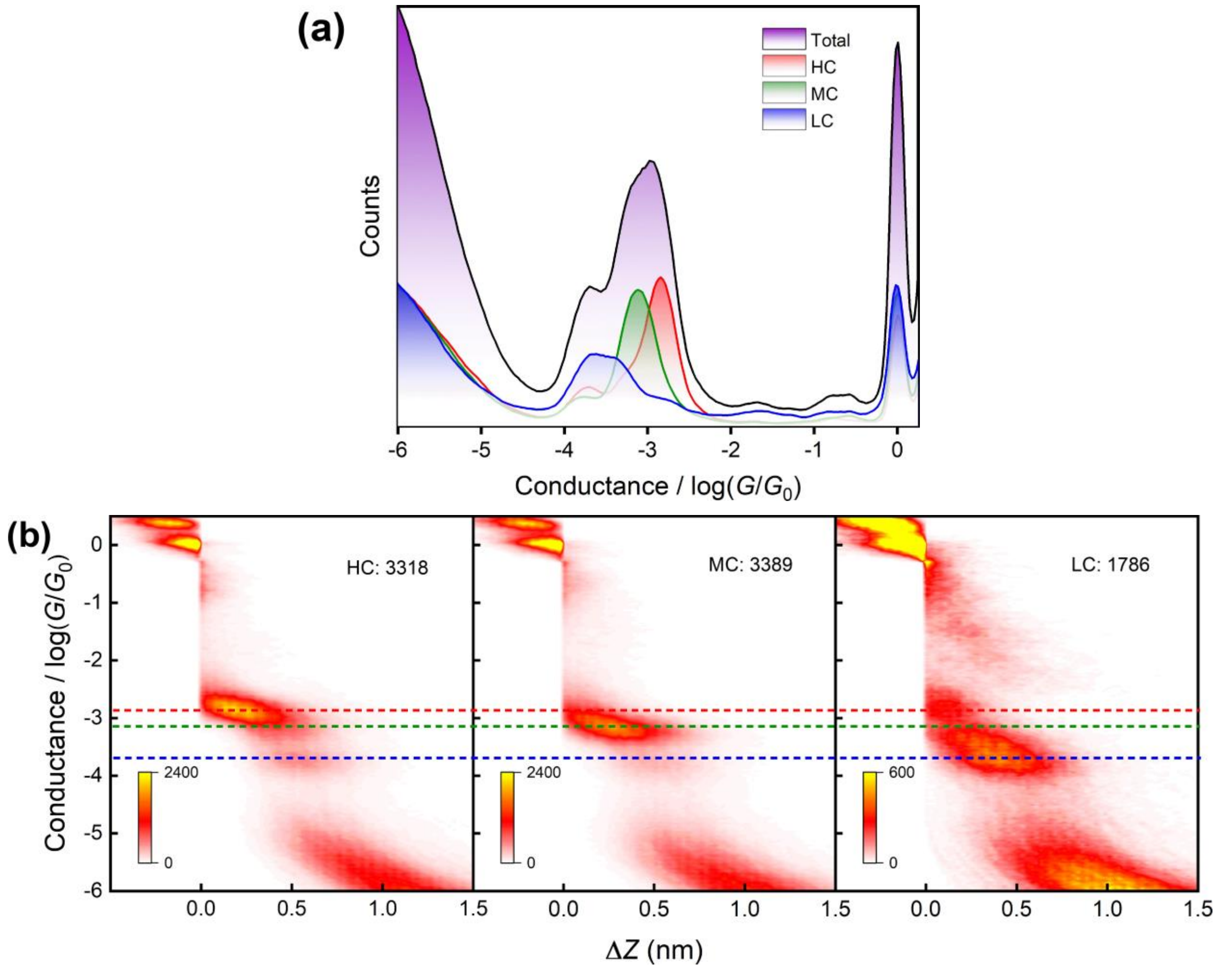

Figure S5. Clustering results of at 4,4'-BPY 10^{-3} M concentration, related to Figure 2. The 1D (a) and 2D (b) conductance histograms of HC, MC, and LC of the 4,4'-BPY molecular junction after clustering at 10^{-3} M concentration.

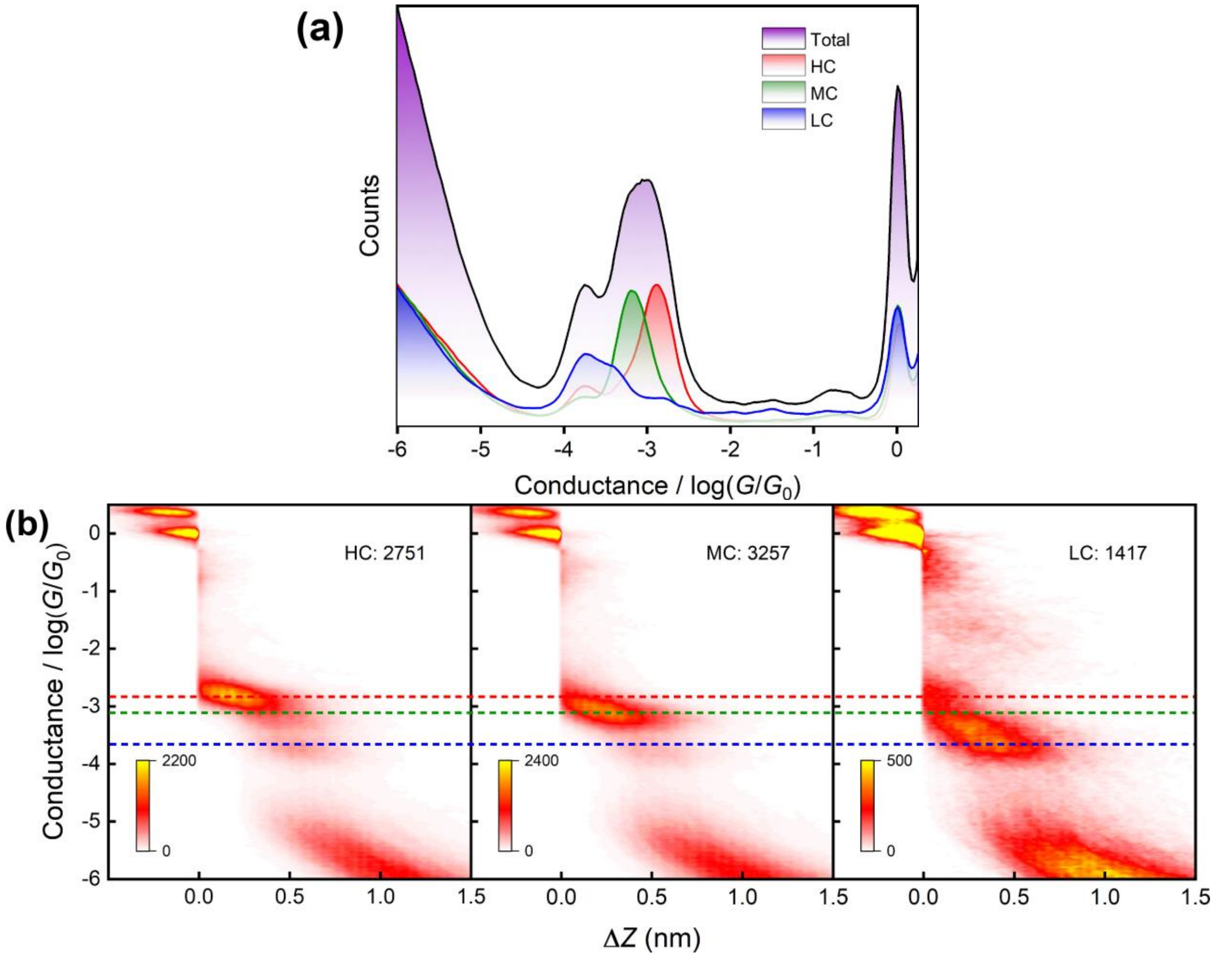

Figure S6. Clustering results of at 4,4'-BPY 10^{-4} M concentration, related to Figure 2. The 1D (a) and 2D (b) conductance histograms of HC, MC, and LC of the 4,4'-BPY molecular junction after clustering at 10^{-4} M concentration.

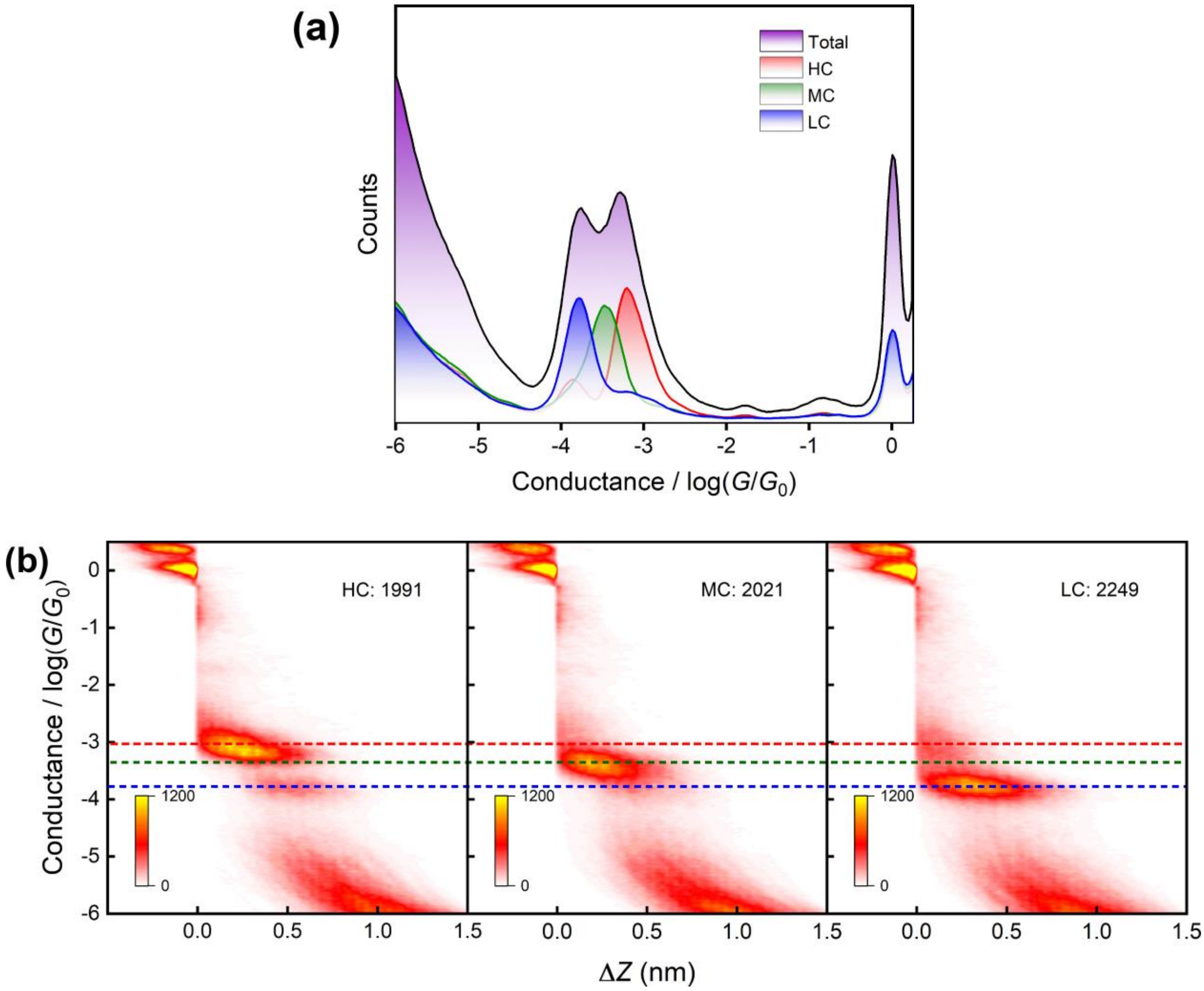

Figure S7. Clustering results of at 4,4'-BPY 10^{-6} M concentration, related to Figure 2. The 1D (a) and 2D (b) conductance histograms of HC, MC, and LC of the 4,4'-BPY molecular junction after clustering at 10^{-6} M concentration.

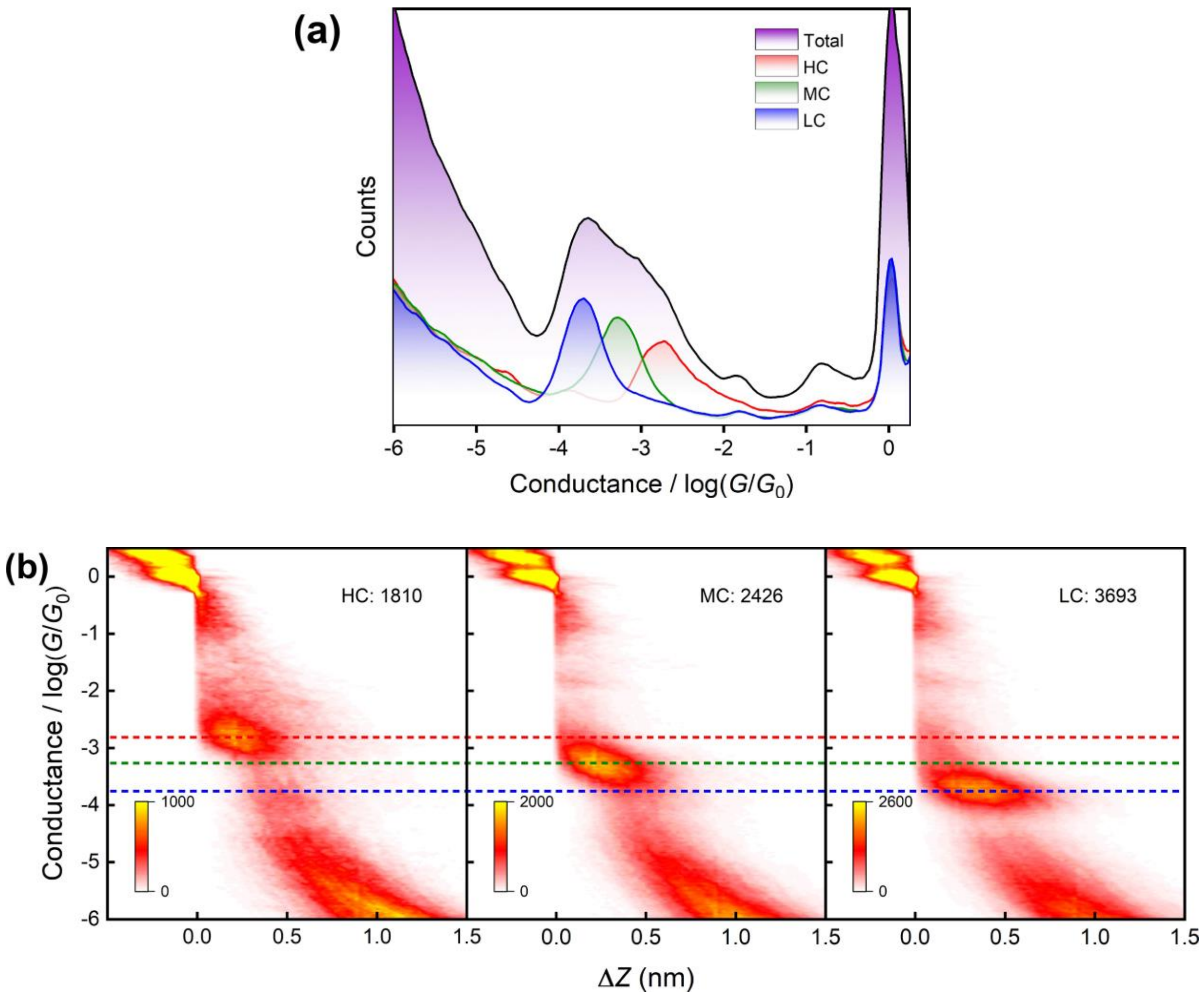

Figure S8. Clustering results of at 4,4'-BPY 10^{-7} M concentration, related to Figure 2. The 1D (a) and 2D (b) conductance histograms of HC, MC, and LC of the 4,4'-BPY molecular junction after clustering at 10^{-7} M concentration.

Table S1. The proportion of HC, MC, and LC of the 4,4'-BPY molecular junction at different concentrations, related to Figure 2.

Concentration	HC	MC	LC
**10^{-3} M**	37.19 ± 1.30 %	43.70 ± 0.65 %	19.11 ± 0.96 %
**10^{-4} M**	38.78 ± 1.10 %	39.87 ± 1.23 %	21.35 ± 0.91 %
**10^{-5} M**	36.70 ± 1.10 %	36.59 ± 0.96 %	26.71 ± 1.40 %
**10^{-6} M**	31.77 ± 1.42 %	32.45 ± 0.95 %	35.78 ± 0.88 %
**10^{-7} M**	22.37 ± 1.32 %	31.07 ± 1.18 %	46.56 ± 0.95 %

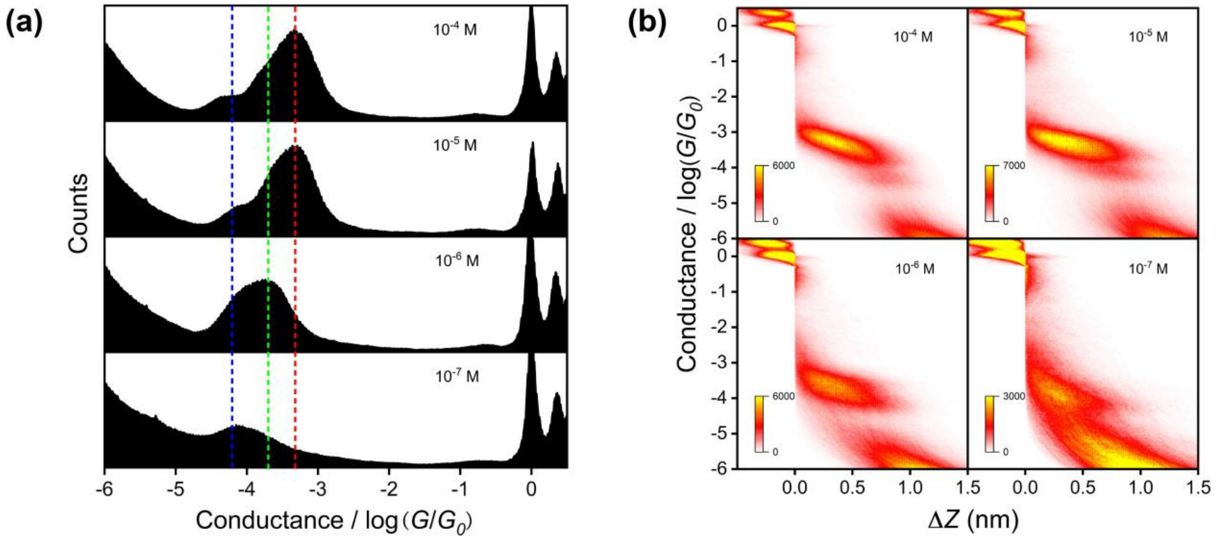

Figure S9. The conductance measurement results of the BPE with different concentrations, related to Figure 2. The 1D (a) and 2D (b) conductance histograms of the BPE (in TCB) molecular junction at the concentrations from 10^{-4} M to 10^{-7} M.

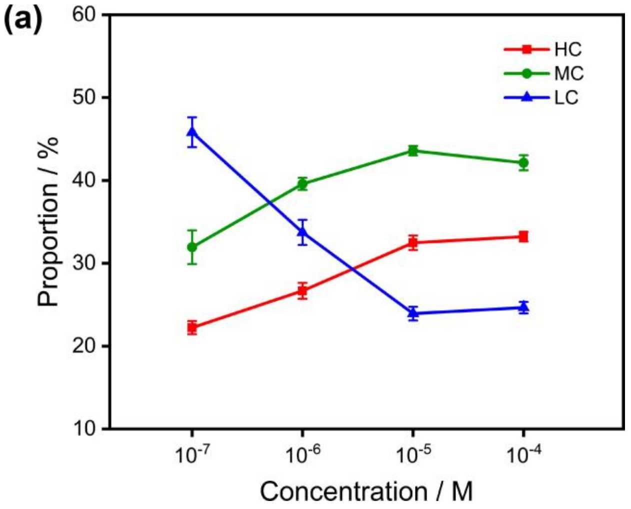

Figure S10. The relationship between three conductance states (HC, MC, and LC) of BPE molecular junction and concentrations, related to Figure 2. The error bars were obtained by three parallel experiments.

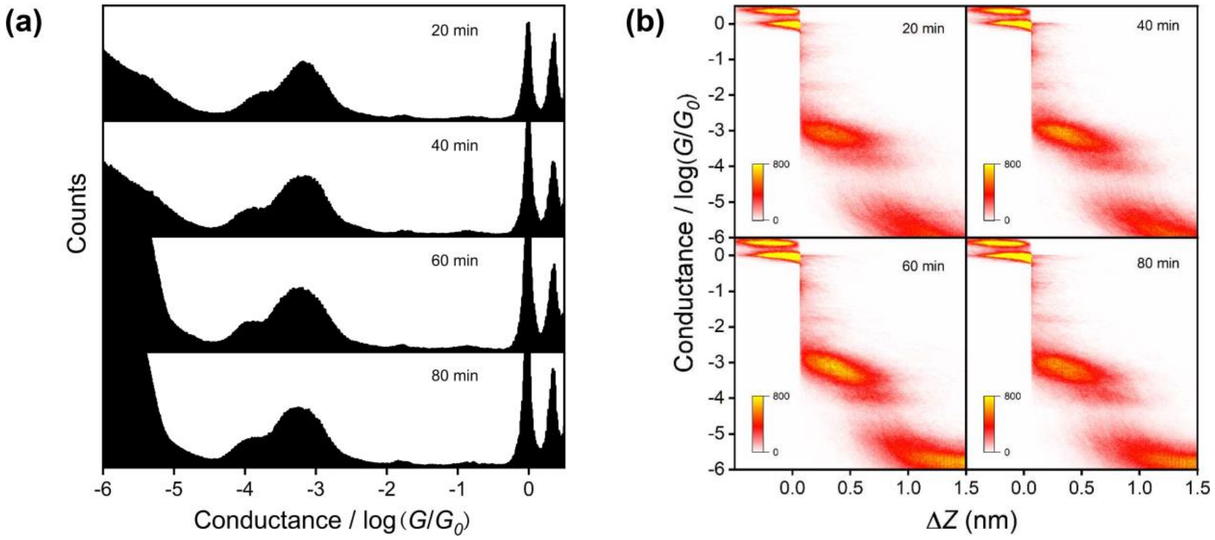

Figure S11. The conductance of 4,4'-BPY in TMB and THF mixed solution. The 1D (a) and 2D (b) conductance histograms of 4,4'-BPY in TMB and THF [V(TMB): V(THF)=4:1] mixed solution at 10^{-4} M concentration.

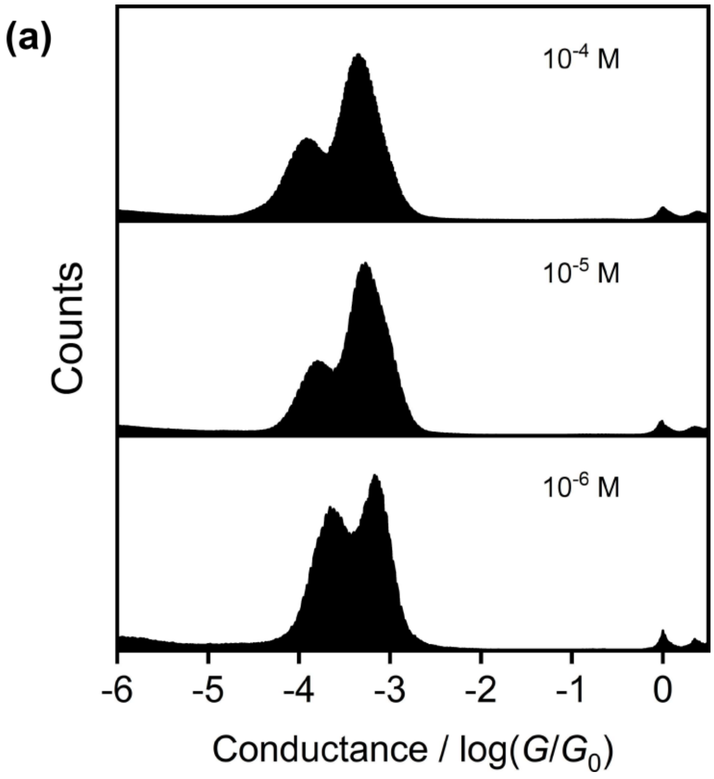

Figure S12. The hovering 1D conductance histogram of the 4,4'-BPY molecular junction at 10^{-4} M, 10^{-5} M, and 10^{-6} M concentration, related to Figure 3.

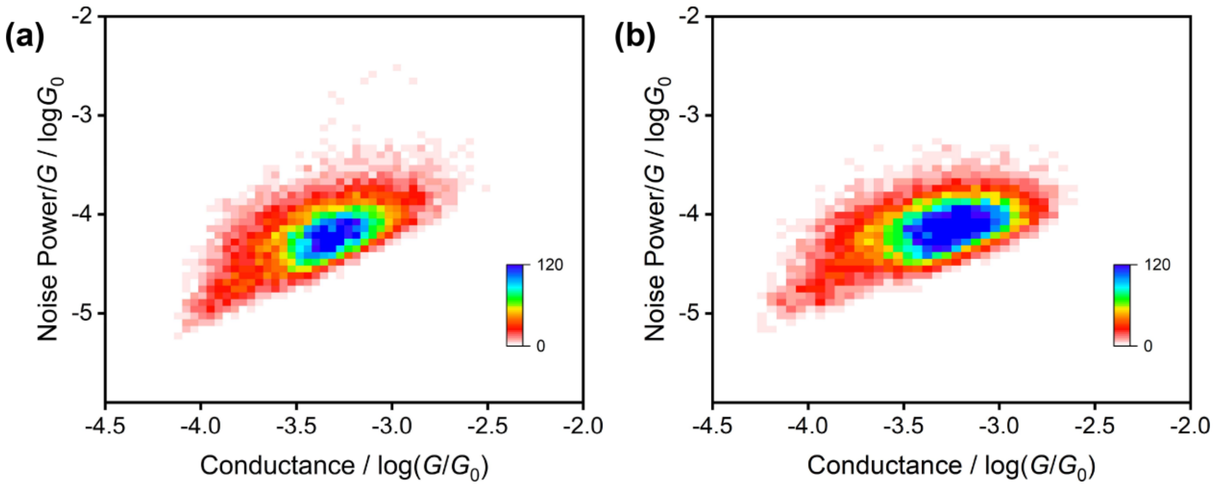

Figure S13. The normalized 2D histogram of the relationship between PSD and conductance for 4,4'-BPY, related to Figure 3. At 10^{-4} (a) and 10^{-5} M (b) concentration, is obtained from 20664 and 21041 traces respectively.

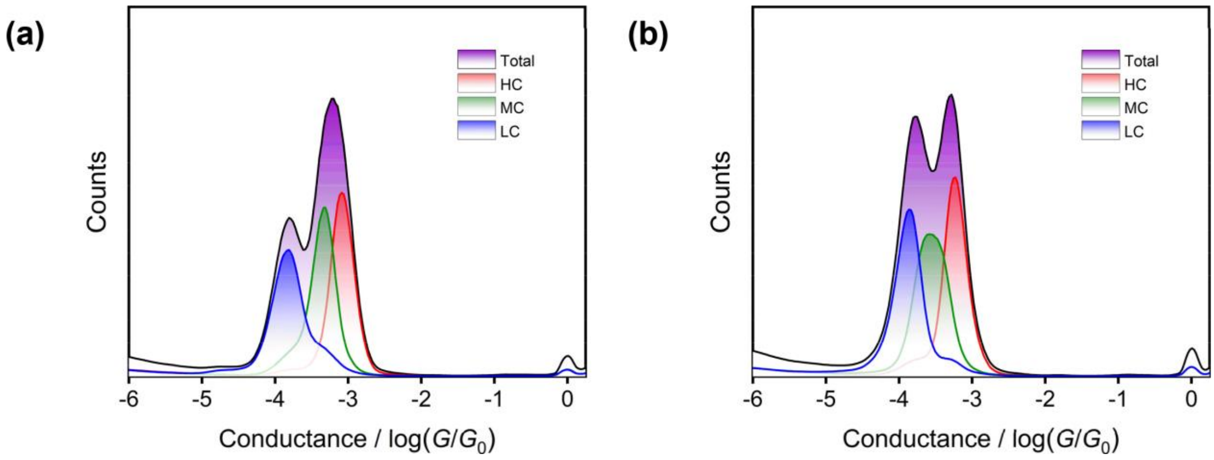

Figure S14. The 1D hover conductance histograms of HC, MC, and LC of the 4,4'-BPY molecular junction after clustering, related to Figure 3. At 10^{-5} M (a) and 10^{-6} M (b) concentration.

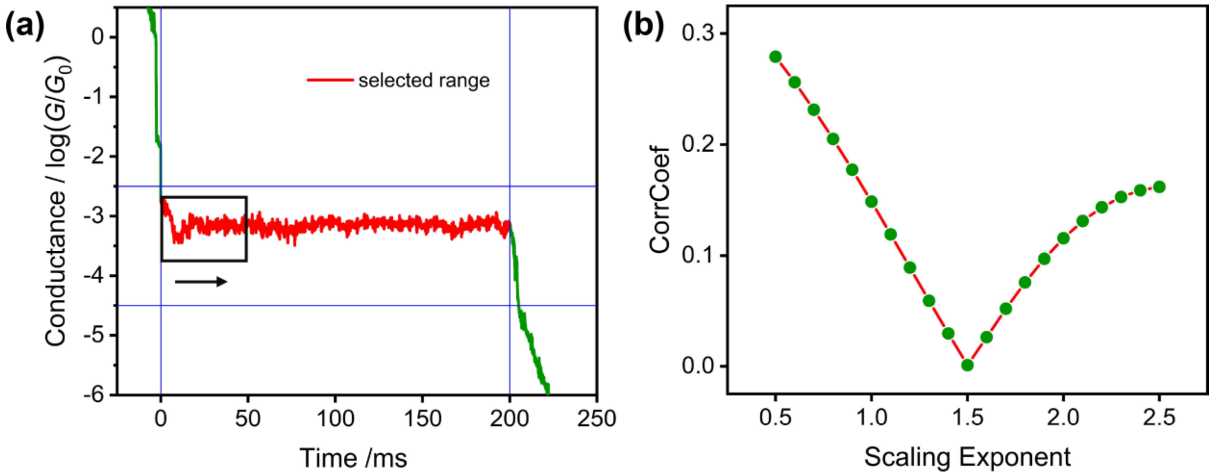

Figure S15. The process of detailed hover analysis, related to Figure 3. (a) Typical 4,4'-BPY molecular junction hover data and data processing selection range. (b) Searching of the scaling exponent leads to the independent of flicker noise power with G_{avg} (the Pearson correlation coefficient approximate to zero) within the range of [0.5, 2.5] with the searching step = 0.1.

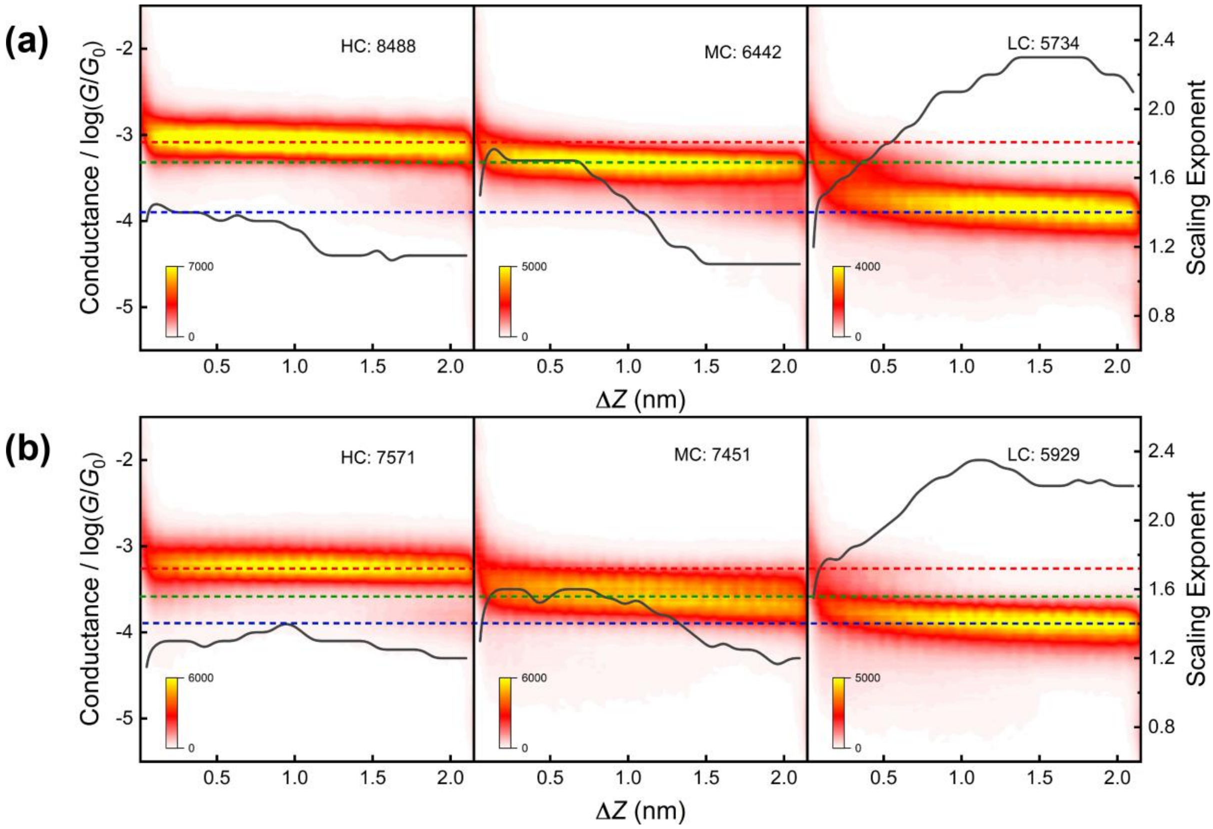

Figure S16. The exponent curve (black solid line) and 2D hover conductance histograms of HC, MC, and LC of the 4,4'-BPY molecular junction after clustering, related to Figure 3. At 10^{-5} M (a) and 10^{-6} M (b) concentration.

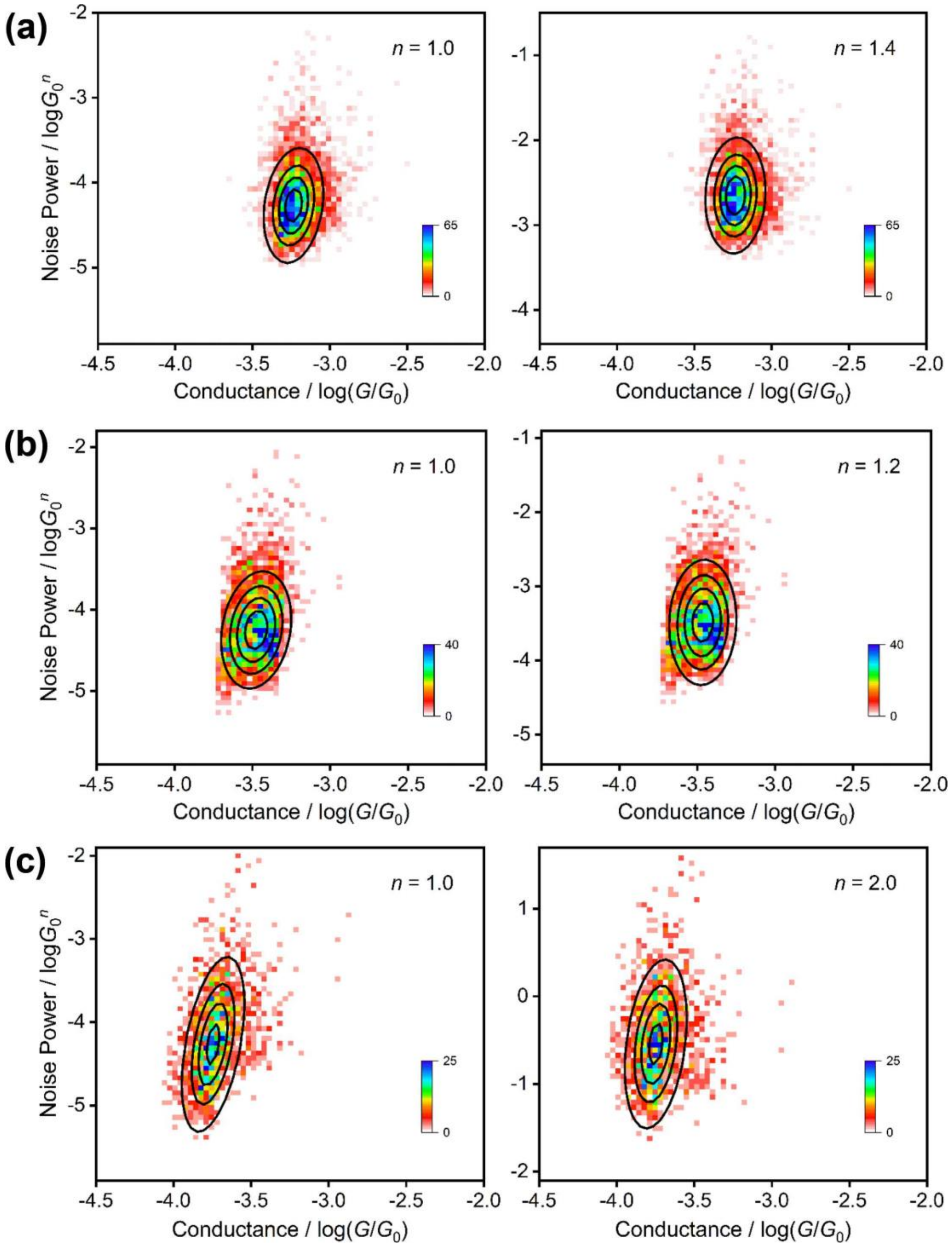

Figure S17. The normalized 2D histogram of the relationship between PSD and conductance for HC (a), MC (b), and LC (c) conductance after clustering in 10^{-4} M, related to Figure 3. HC, MC, and LC are obtained from 9253, 5321, and 5538 traces respectively. Dotted contours represent fits to the bivariate normal distribution.

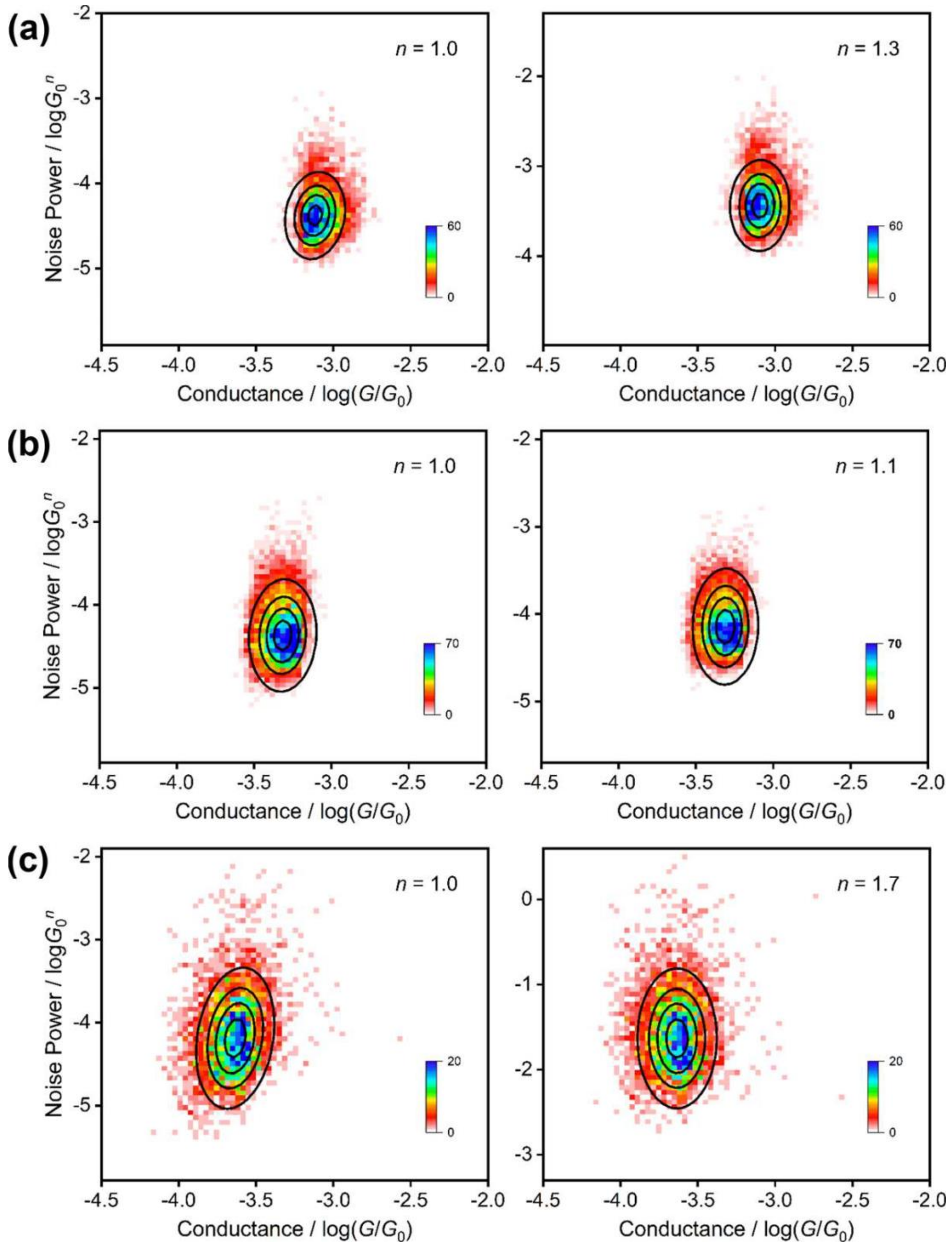

Figure S18. The normalized 2D histogram of the relationship between PSD and conductance for HC (a), MC (b), and LC (c) conductance after clustering in 10^{-5} M, related to Figure 3. HC, MC, and LC are obtained from 8488, 6442, and 5734 traces respectively. Dotted contours represent fits to the bivariate normal distribution.

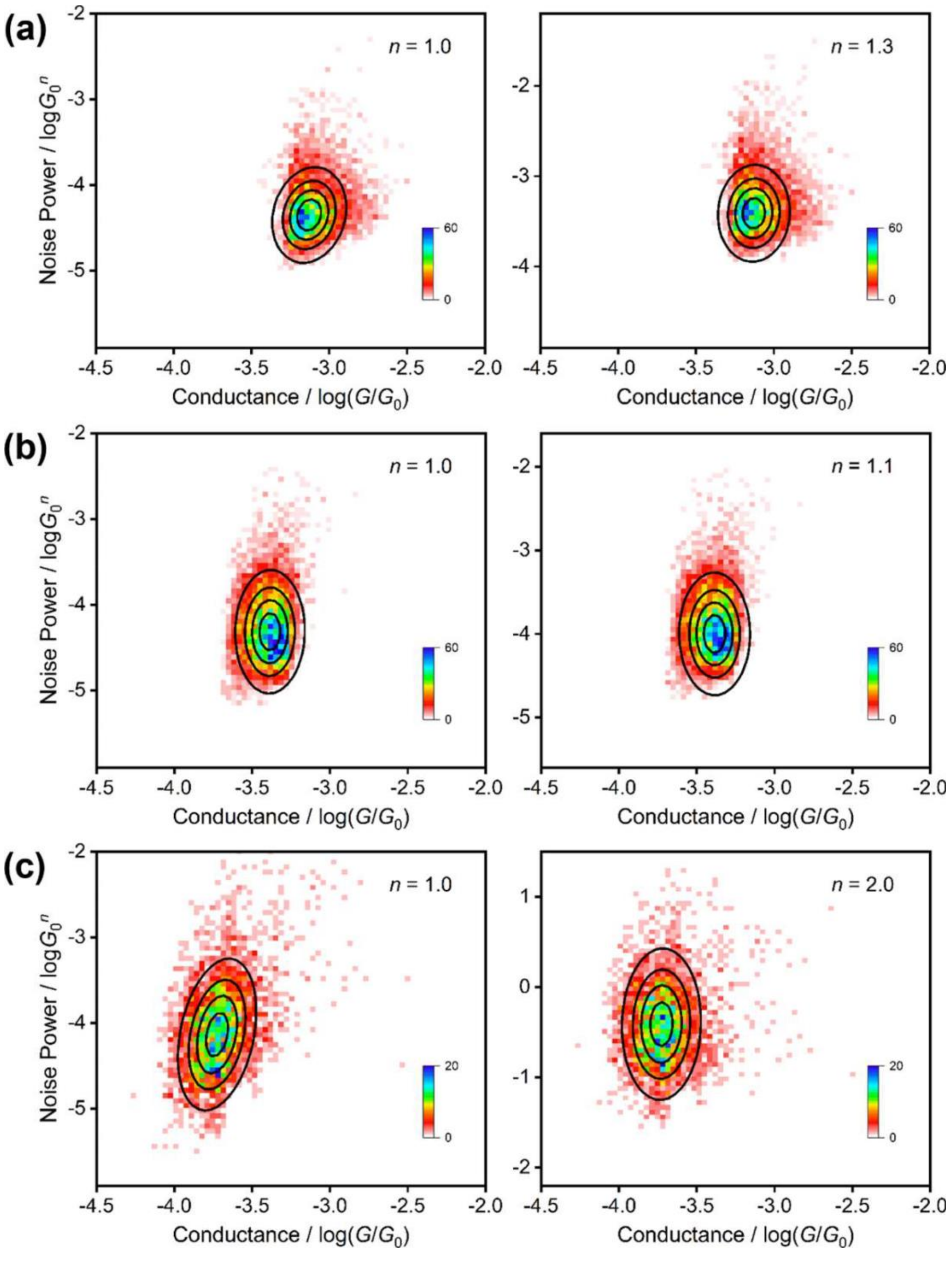

Figure S19. The normalized 2D histogram of the relationship between PSD and conductance for HC (a), MC (b), and LC (c) conductance after clustering in 10^{-6} M, related to Figure 3. HC, MC, and LC are obtained from 7571, 7451, and 5929 traces respectively. Dotted contours represent fits to the bivariate normal distribution.

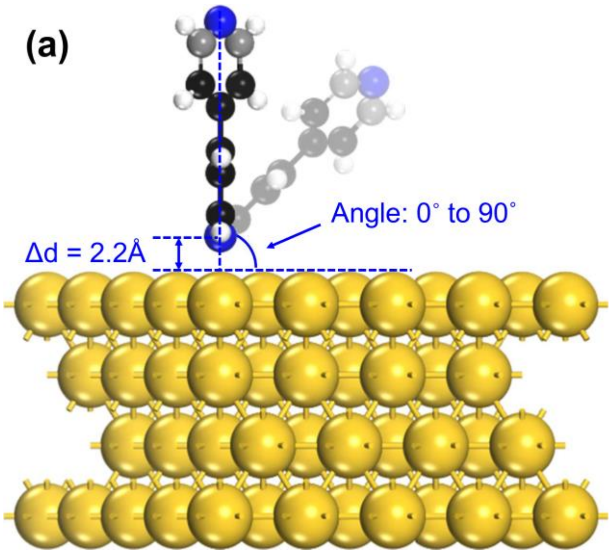

Figure S20. Structural optimization diagram of 4,4'- BPY molecules adsorbed at different angles on Au(111) surface, related to Figure 4.

Table S2. **The image charge correction (*W*), corrected self-energy (Σ^{LUMO}) and the final position of the LUMO orbital relative to the Fermi energy (Σ) for the three junctions, related to Figure 4.**

	**35°**	**65°**	**90°**
**a / Å**	4.68	4.03	3.20
**W / eV**	1.07	1.23	1.56
**Σ^{LUMO} / eV**	1.94	1.78	1.45
**Σ / eV**	1.16	1.32	1.65

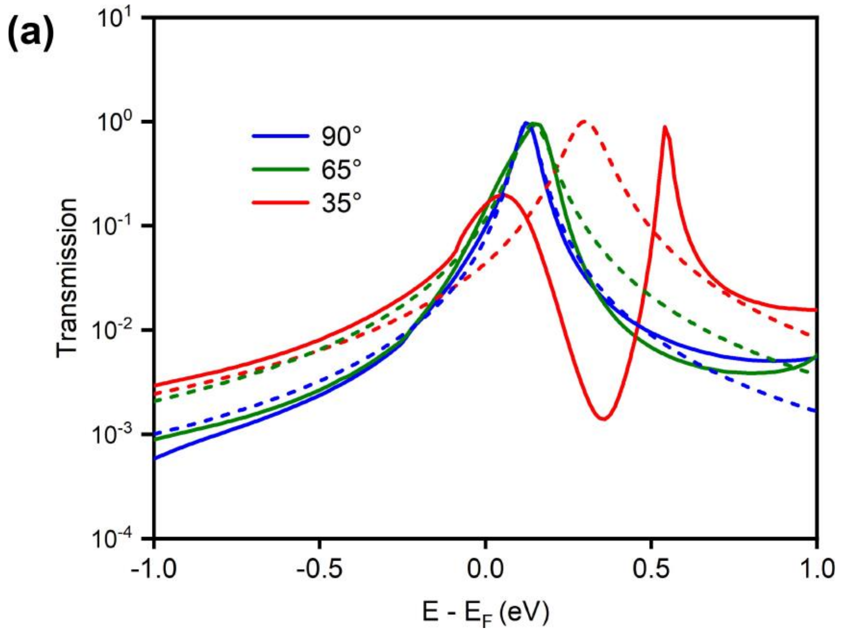

Figure S21. The calculated transmission function for molecular junctions at different angles, related to Figure 4. The solid line represents the calculated curve, and the dotted line represents the curve that is fitted but not corrected (Σ=0), the blue, green, and red dotted line represents 90°, 65°, and 35°, respectively.

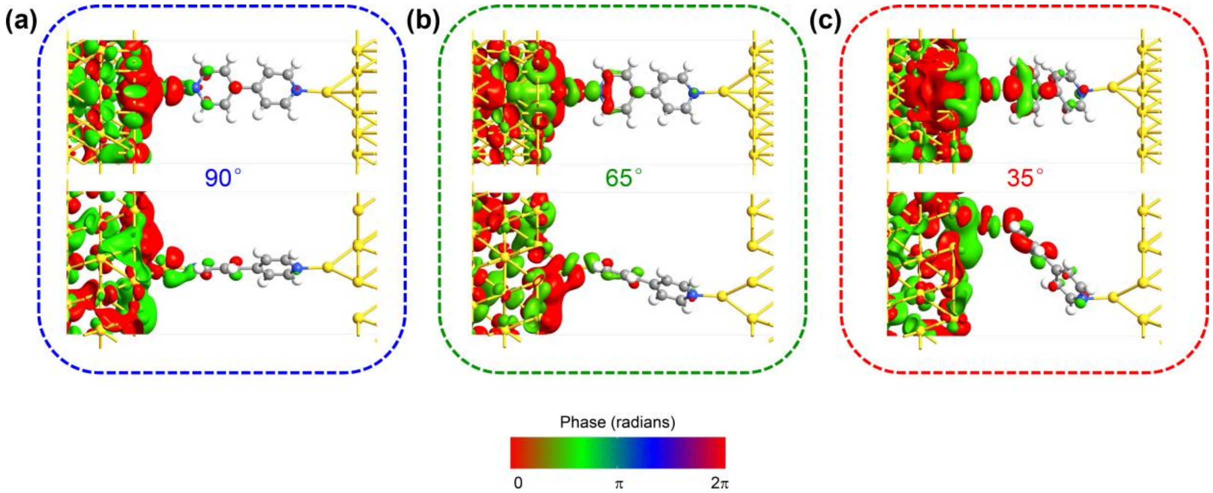

Figure S22. The transmission eigenstates of the 4,4'-BPY molecule junction, related to Figure 4. (a), (b), (c) represent 90°, 65°, and 35° respectively. The above picture is the top view, and the below is the front view.

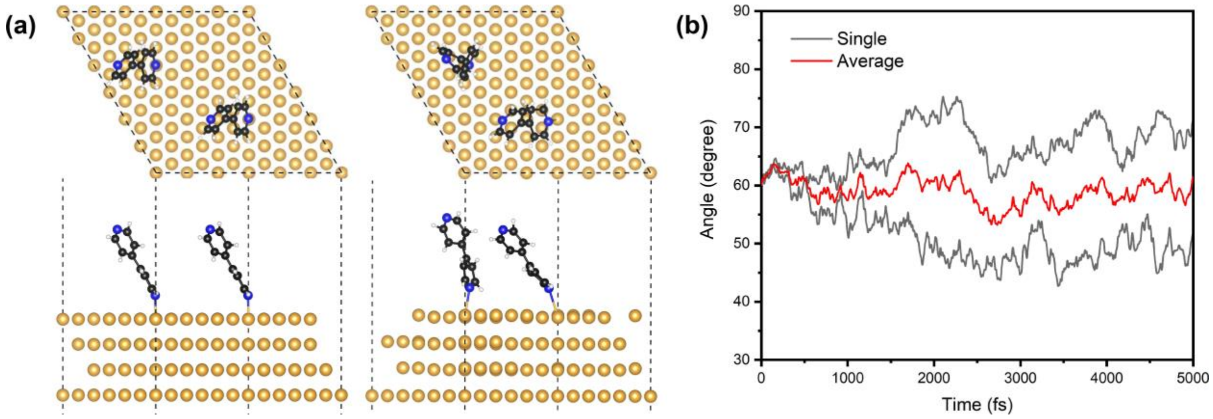

Figure S23. AIMD simulation for C2, related to Figure 4.

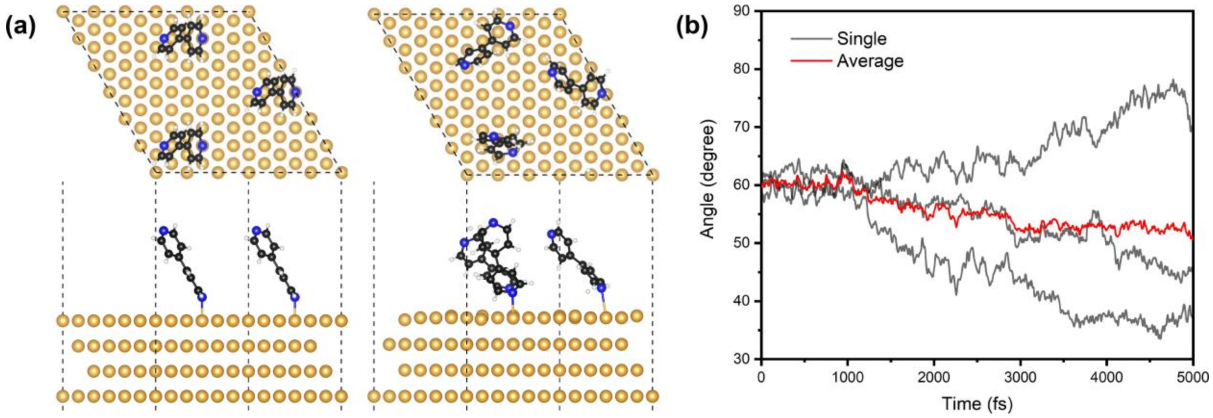

Figure S24. AIMD simulation for C3, related to Figure 4.

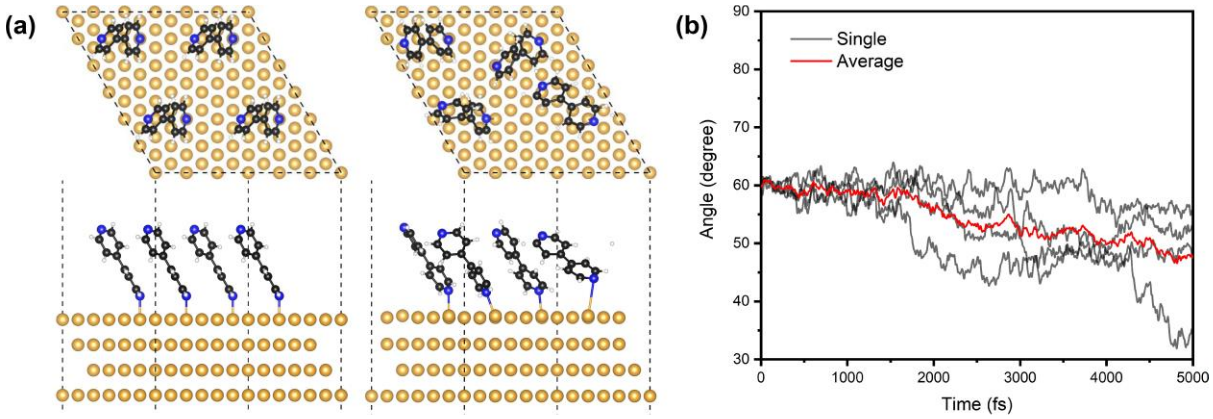

Figure S25. AIMD simulation for C4, , related to Figure 4.
